# Effects of dance on gait and dual-task gait in Parkinson’s disease

**DOI:** 10.1371/journal.pone.0280635

**Published:** 2023-01-24

**Authors:** Nadeesha Kalyani Hewa Haputhanthirige, Karen Sullivan, Gene Moyle, Sandy Brauer, Erica Rose Jeffrey, Graham Kerr

**Affiliations:** 1 Movement Neuroscience Group, Centre for Biomedical Technologies, Queensland University of Technology, Brisbane, Queensland, Australia; 2 Faculty of Health, School of Exercise and Nutrition Sciences, Queensland University of Technology, Brisbane, Queensland, Australia; 3 Faculty of Medicine, Department of Allied Health Sciences, University of Colombo, Sri Lanka; 4 Faculty of Health, School of Psychology and Counselling, Queensland University of Technology, Brisbane, Queensland, Australia; 5 Faculty of Creative Industries, School of Creative Practice, Queensland University of Technology, Brisbane, Queensland, Australia; 6 Academic Division, Office of the Deputy Vice-Chancellor (Academic), Queensland University of Technology, Brisbane, Queensland, Australia; 7 Faculty of Health and Behavioural Sciences, School of Health and Rehabilitation Sciences, University of Queensland, Brisbane, Queensland, Australia; 8 Queensland Ballet, South Brisbane, Queensland, Australia; 9 Dance for Parkinson’s Australia, Brisbane, Queensland, Australia; La Trobe University, AUSTRALIA

## Abstract

**Background:**

Gait impairments in Parkinson’s disease (PD) limit independence and quality of life. While dance-based interventions could improve gait, further studies are needed to determine if the benefits generalise to different terrains and when dual-tasking. The aim was to assess the effects of a dance intervention, based on the Dance for PD^®^ (DfPD^®^) program, on gait under different dual-tasks (verbal fluency, serial subtraction) and surfaces (even, uneven), and to determine if a larger scale follow-up RCT is warranted.

**Methods:**

A dance group (DG; n = 17; age = 65.8 ± 11.7 years) and a control group (CG: n = 16; age = 67.0 ± 7.7 years) comprised of non-cognitively impaired (Addenbrooke’s score: DG = 93.2 ± 3.6, CG = 92.6 ± 4.3) independently locomoting people with PD (Hoehn & Yahr I-III). The DG undertook a one-hour DfPD®-based class, twice weekly for 12 weeks. The CG had treatment as usual. The spatiotemporal variables of gait were assessed at baseline and post-intervention while walking on two surfaces (even, uneven) under three conditions: regular walking; dual-task: verbal-fluency (DT_VERB_), and serial-subtraction (DT_SUBT_). The data were analysed by means of a linear mixed model.

**Results:**

At baseline, there was no significant group difference for any spatiotemporal gait variable. The DG improved significantly compared to the CG with and without a dual task when walking on even surface. During regular walking, DG improved in gait velocity (p = 0.017), cadence (p = 0.039), step length (p = 0.040) and stride length (p = 0.041). During DT_VERB_ significant improvements were noted in gait velocity (p = 0.035), cadence (p = 0.034) and step length (p = 0.039). The DG also exhibited significant improvement compared to the CG during DT_SUBT_ in the measures of gait velocity (p = 0.012), cadence (p = 0.021), step length (p = 0.018), and stride length (p = 0.151).

On the uneven surface, improvements were noted when walking while performing serial subtractions only. During regular walking, improvements were noted for the CG but not for the DG. CG has spent less time in double support following the intervention than DG. While DT_VERB_ condition had no significant group differences for any gait parameter (p’s >0.05), in the DT_SUBT_ condition, the DG improved significantly compared to the controls on gait velocity (p = 0.048), cadence (p = 0.026), and step length (p = 0.051).

**Conclusions:**

DfPD®-based classes produced clinically significant improvement in spatiotemporal gait parameters under dual-task conditions and on uneven surfaces. This could arise from improved movement confidence and coordination; emotional expression; cognitive skills (planning, multitasking), and; utilisation of external movement cues. A large-scale RCT of this program is warranted.

**Trial registration:**

A protocol for this study has been registered retrospectively at the Australian New Zealand Clinical Trials Registry. Identifier: ACTRN12618001834246.

## Introduction

Improving gait is of fundamental importance for people with Parkinson’s disease (PD) as it is associated with independence and quality of life [1–4]. Amidst many debilitating symptoms, gait disturbances are a frequent cause of disability, and are characterised by reduced gait velocity, cadence, stride length, swing time, and arm swing and a consequent increase in the double support phase compared to age-matched healthy controls [1,3,4]. These deficits are also associated with high fall risk [5]. Increased muscle rigidity reduces body rotation and encourages abnormal head-trunk intersegmental coordination during walking, thereby affecting gait velocity and turning [6,7]. Stride time variability, which is a marker for impaired mobility [8], is increased. Given the wide range of potential PD-related gait disturbances and associated functional impairments, it is vital to find effective gait improvement programs.

An effective gait improvement program for people with PD should show benefits in a variety of real-world conditions. For example, improvements should be evident when walking *and* talking (dual-task gait) and when walking on different terrains (e.g. on an uneven surface). People with PD are known to have dual-task gait deficits [9]. This is thought to be because dual-tasking relies on executive function and the ability to divide attention [10], both of which are affected by basal ganglia pathology even in early PD [11]. The additional attentional demands of walking on uneven surfaces could also explain why this ability is compromised in many people living with PD [8,12]. Therefore, a gait intervention that improves walking on varied terrains and under dual-task conditions should facilitate ambulation for people with PD.

Dance is a complex sensorimotor rhythmic activity that involves stepping across the floor in a variety of ways including heel-toe and toe-heel walking, gliding, marching, waltz, and combinations of these [13]. Different dance types such as Tango, American Ballroom, and contact improvisation have exhibited positive effects on gait in people with PD [14–19]. DfPD^®^ (Dance for Parkinson’s disease^®^) methodology, which incorporates different dance types, has also demonstrated beneficial effects [19,20]. Dance contains different characteristics such as musicality (dancing to the instrumental rhythms), synchronized movements, and cueing strategies which may lead to gait improvement [21–25]. It was hypothesised that various external movement cues (visual, auditory, and somatosensory) may bypass the defective basal ganglia in individuals with PD by using alternative pathways [26]. These cues may help to initiate, sustain and terminate movements [13]. Visual feedback is received by seeing the performance of the dance teachers. Rhythmic auditory cues and attentional strategies are equally effective in improving walking speed and step amplitude [21]. Music used in dance is an important auditory cue that can help maintain rhythm and unity among the dancers [22]. In improvisational dance auditory cues and aesthetic imagery are used to convey improvisational ideas that provoke novel stationary and locomotor movement from the dancers [22]. These auditory cues improve gait by acting as external signals for the temporal processing and internal rhythmic timing which is impaired in PD [27]. Participants receive sensory input when holding hands with partners in a circle or line. This light touch acts as a somatosensory cue to focus attention on balance [20]. Dance also incorporates elements of balance exercises, which in turn facilitates walking, both strengthening, such as standing on one leg, as well as partner dancing where one must control balance dynamically and respond to perturbations within the environment [28].

Dance also improves dual tasking. Dancers need to execute the steps, and navigate among other dancers across the floor while listening to music [29]. Also, when dancing, one must maintain balance against the perturbations in the environment, while engaged in complicated hand and foot coordination patterns. All these dual tasks require attention. Dancers need to remember a number of dance routines, to rapidly and skilfully switch between them [14]. Therefore, dancing has helpful effects on selected cognitive skills [30]. Particularly, the effects of executive function and episodic memory may have beneficial effects on dual-task gait. These key features of dance, which can be considered similar to traditional exercise interventions, make dance therapeutically meaningful and are consequently speculated as a plausible alternate management option for people with PD.

In a recent systematic review and meta-analysis, we found promising findings for gait improvement in people with PD following dance-based interventions [31]. Improvements were shown in measures of functional mobility including the Timed Up and Go Test (TUG) [13,32–37] and the Six-Minute Walk Test (6MWT) [32–34,38]. Eight studies looked for dual-task gait improvement from a dance-based intervention [13,22,33,36–40]. Only one dance style (Argentine Tango) showed a dual-task gait benefit on the TUG [33,37,38]. Further, a limited number of studies, mostly using Argentine Tango [13,17,33,38,41–44], have examined effects using *spatiotemporal gait variables* such as velocity, cadence, swing, stride, and double support percentage [13,17,33,38,40–45], and only one of these parameters (gait velocity) has been tested whilst dual-tasking [13,38]. There have been mixed findings from dance-based interventions with these finer-grained gait measures; however, three studies demonstrated a benefit on selected parameters [cadence, stride length while walking backward, swing, and stance percentage; [34,39,45]. Further exploration of the potential benefits of the gait of dance-based interventions for people with PD is required because there has not yet been a detailed spatiotemporal gait analysis under dual-task conditions following these dance interventions [31], and it has yet to be established if the benefits extend to uneven surfaces.

This study investigated the effects on spatiotemporal measures of gait (regular and dual-task) of an intervention modelled on an internationally adopted DfPD^®^ program. In 2001, the Brooklyn Parkinson Group and Mark Morris Dance Group in New York, took the initial step in the development of the DfPD^®^ programme for people with PD and caregivers, in order to experience the joyfulness of dance and possible motor and cognitive benefits for the people with PD [20]. The DfPD^®^ methodology produces a complex multifaceted intervention. It includes music, movement, social and artistic elements, and it has a central component that draws on different dance styles. These program attributes theoretically enable it to produce more benefits than simpler programs or those based on a single dance style. Empirical studies of the DfPD^®^ approach have already shown that it benefits a wide range of outcomes, including quality of life [46] and functional gait [19,20]. It remains to be determined if this approach produces measurable benefits on spatiotemporal gait parameters, under dual-task conditions, and on different surfaces. Spatiotemporal measurement of gait provides a direct measure of the *components* of walking, and this could provide insight into the mechanism for change in the functional measures. We hypothesized that, compared to a control condition, there would be an improvement in regular and dual-task gait on even and uneven surfaces following a dance-based intervention.

### Methods

This study incorporated a quasi-experimental parallel group pre-test, post-test design and was carried out at the Institute of Health and Biomedical Innovation, Queensland University of Technology (QUT). Participants gave written informed consent under the Declaration of Helsinki [47] and the experimental protocol was approved by the Human Research Ethics Committee at QUT (#1700000005). The study was registered retrospectively in the Australian New Zealand Clinical Trials Registry (ANZCTR) (#ACTRN12618001834246).

### Participants

A sample size of 16 participants per group was estimated-based on parameters drawn from a previous pilot study [48] (alpha = 0.05, power = 0.8, effect size = 0.75). The study was powered on the effects of the dual task Timed Up and Go (TUG) test. Dual task TUG assesses cognitive dual task performance during gait [49]. Participants were recruited from PD support groups in Queensland, advertising on the Parkinson’s Queensland website, distributing flyers to participants in the existing DfPD^®^ class at Queensland Ballet (QB), through the “Radio Parkies” radio show, and via the QUT email system.

Forty-six people with PD expressed interest in the study and were interviewed by phone to determine their eligibility. Eligible participants: 1) required a clinical diagnosis of idiopathic PD, using the published diagnostic criteria for clinically defined *definite PD* [50]; 2) were aged between 40–85 years, which was the typical age range of participants recruited in many previous studies and is typical of the PD population [30,51]; 3) had mild to moderate stage disease (Hoehn and Yahr I-III) [52]; 4) were free from dementia (Addenbrooke’s Cognitive Examination: ACE) > 82) [53]; 5) had no medical, neurological (other than PD), musculoskeletal, cardiovascular or respiratory abnormalities, and; 6) could walk independently for ≥ 3 m without an assistive device. Participants were on stable medication regimens and were tested in the ‘‘ON” medication phase (within three hours post-PD medication).

**Fig 1:**
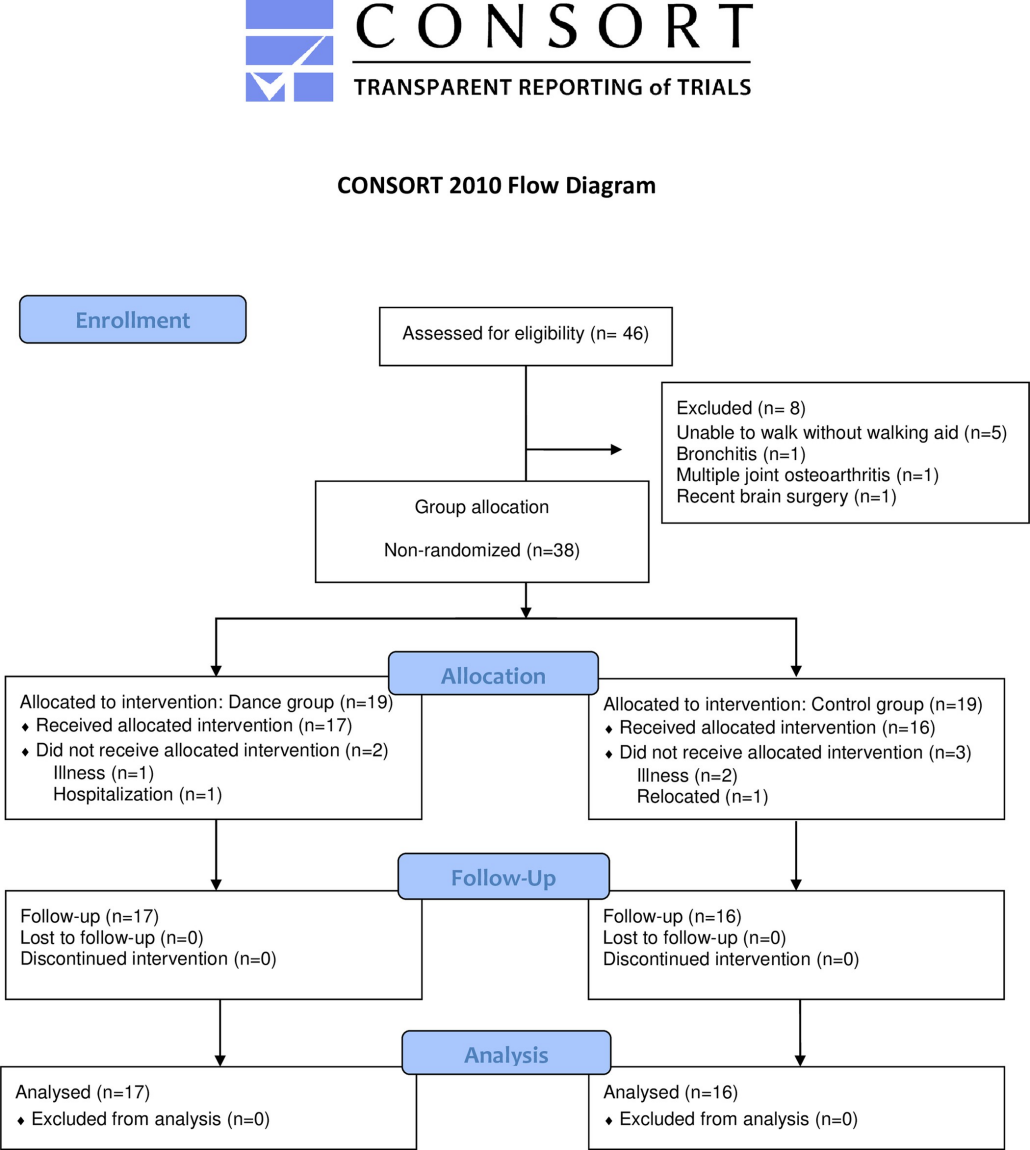
Consort diagram of participant recruitment, group allocation, and tracking over the course of the study.

Following an initial screening, 38 people were deemed eligible. Initially, a blinded allocation to dance and control groups was carried out. While two-thirds of participants were randomly allocated via this method, due to recruitment difficulties, the study timeframe, and participant availability, the remaining participants were allocated based on their preference. Therefore, the group allocation was pseudo-random (dance group: DG = 19 and control group: CG = 19). During baseline assessment, two DG participants and three CG participants discontinued, leaving 17 and 16 participants in the DG and CG, respectively (Fig 1).

### Baseline assessment

Baseline assessment included assessing the disease severity, level of cognition, falls, and medication history (Table 1). The Hoehn and Yahr Scale (H&Y scale), Movement Disorder Society Unified Parkinson’s Disease Rating Scale (MDS-UPDRS), and Addenbrooke’s Cognitive Examination (ACE) were used as screening tools.

**Table 1:** Descriptive statistics and group comparisons of baseline and PD characteristics.

Characteristics	Dance group (n = 17)	Control group (n = 16)	*p*-value
Gender, n (%) Male Female	3 (17.6%) 14 (82.4%)	10(62.5%) 6(37.5%)	**0.013**
Age (years), M(SD)	65.24 ± 11.88	66.50 ± 7.70	0.721
Education, n (%) Primary school Secondary school Technical college University	0 (0%) 5 (29.4%) 1 (5.9%) 11 (64.7%)	0 (0%) 8 (50%) 3 (18.8%) 5 (31.3%)	0.180
Number of falls during previous six months, M (SD)	0.18 ± 0.39	0.13 ± 0.34	0.692
Years since PD diagnosis, M (SD)	3.76 ± 2.88	5.94 ± 3.61	0.064
Levodopa equivalent dose (mg/day) M (SD)	555.57 ± 332.41	715.56 ± 418.38	0.261
MDS-UPDRS, M (SD)	73.82 ± 25.67	58.06 ± 22.72	0.072
MDS-UPDRS motor subscale III, M (SD)	38.71 ± 17.67	30.25 ± 15.64	0.157
Hoehn and Yahr scale, M (SD)	1.65 ± 0.79	1.56 ± 0.81	0.763
Addenbrooke’s examination, M (SD)	93.71 ± 3.18	92.69 ± 4.33	0.445

*M* = mean, *SD* = standard deviation: Comparisons are performed with either independent samples *t-test* or Fisher’s exact test whenever appropriate. MDS-UPDRS: Movement Disorder Society: Unified Parkinson’s Disease Rating Scale. Bold values are significant (i.e., *p*-value < 0.05).

The H&Y scale is a simple and descriptive staging scale developed to evaluate the clinical function of PD [54]. This is a five-point scale which was designed on the basis that the severity level of PD relates to bilateral motor involvement and balance/gait dysfunctions. The current study included participants with mild to moderate disease severity levels which included stages from 1–3 on the H&Y scale. Stage 1 is the mildest form of PD which the symptoms are mild and not enough to interfere with overall lifestyle. Stage 2 is considered a moderate form of the disease and the symptoms are relatively more noticeable than stage 1. While muscle stiffness prolongs task completion, stage 2 does not impair balance. Gait difficulties may develop, however, the person can still live independently. The middle stage in PD is stage 3 and the people are more likely to experience balance and gait impairments but are ambulatory and are still able to complete daily tasks.

The MDS-UPDRS was used as an index of the disease-related symptom severity. MDS-UPDRS is one of the three most evaluated, valid, and reliable instruments for the assessment of PD and is the most extensively used scale in research studying PD [55]. Each parkinsonian sign or symptom is rated on a 5-point Likert-type scale (ranging from 0 to 4). MDS-UPDRS is divided into four parts and the part III, which is the Motor Examination includes the severity of PD gait impairment. It consists of 33 items and the maximum score of 132 indicates the worst motor severity.

Addenbrooke’s Cognitive Examination (Australian Version) was used to assess overall cognition and screen for dementia [56]. It covers five cognitive domains (attention/ orientation, memory, verbal fluency, language, and visuospatial ability), is validated for PD and is sensitive to detecting early cognitive dysfunction and has established reliability and validity in evaluating dementia. the ACE screening, participants with a score <82 were included in the study.

### Spatiotemporal gait analysis

The spatiotemporal gait assessment used a 12-camera Vicon data capturing system (Cameras: Vantage 5, 200Hz; Software: Nexus 2.5). The data were captured from forty-one retro-reflective markers worn by the participant while they walked from start to finish along a 12m walkway.

The reflective markers were bilaterally attached to the skin surface on the anatomic points according to the Helen Hayes marker set [57]. A headband with four markers attached was placed around the forehead. The markers were positioned over the right and left temples anteriorly. The posterior markers of the head were positioned roughly horizontal plane to the frontal markers. The main anatomical landmarks for fixing the markers in the trunk were the jugular notch, xiphoid process, C7 spinous process, middle of the right scapular, T10 spinous process, anterior superior iliac spines, and posterior superior iliac spines. In the upper limb segment, the markers were mainly placed on the acromion process, lateral epicondyle, radial and ulnar styloid processors, and dorsum of the hand below the head of the 2^nd^ metacarpal. The prominent markers for the lower limb segment were lateral femoral condyle, lateral malleolus, on calcaneus at the same height above the plantar surface of the foot marker and 2^nd^ metatarsal head.

Marker positions were tracked within a central six-meter length for four complete gait cycles. Data processing included marker labelling, reconstructing, gap filling, modelling, and inserting the events (heel strike, toe off) for each walking trial [58]. Marker trajectories were filtered using a Woltring generalized cross-validatory interpolating spline (5^th^ order) [59]. To enable the calculation of spatiotemporal data, it was necessary to insert events in the gait cycle that corresponded to when the foot contacted (heel strike) and left the ground (toe off).

Gait velocity was the primary outcome. Secondary outcomes were cadence, step length, stride length, double support %, stance phase %, swing phase %, single support %, and stride time variability. These gait parameters were calculated for each gait cycle in the following manner: (1) Gait velocity (ms^-1^) [distance covered between the first and last footfalls divided by the elapsed time], (2) Cadence (steps/second) [the number of steps taken in a second], (3) step length (metres) [Distance between two consecutive contacts of feet (heel-heel)], (4) Stride length (metres) [distance covered by the left or right heel between two consecutive heel contacts with the same foot], (5) double support % [100 x double stance time/ stride time], (6) stance phase % [100 x stance time/stride time], (7) swing phase % [100 x swing time/stride time], (8) single support % [100 x single support time/stride time], (9) Stride time variability [fluctuation in gait stride time between steps and was measured using the standard deviation of the stride time] [60]. Some of these variables were directly exported from the software and custom software was used to calculate the remaining variables.

### Walking conditions and surfaces

**Fig 2:**
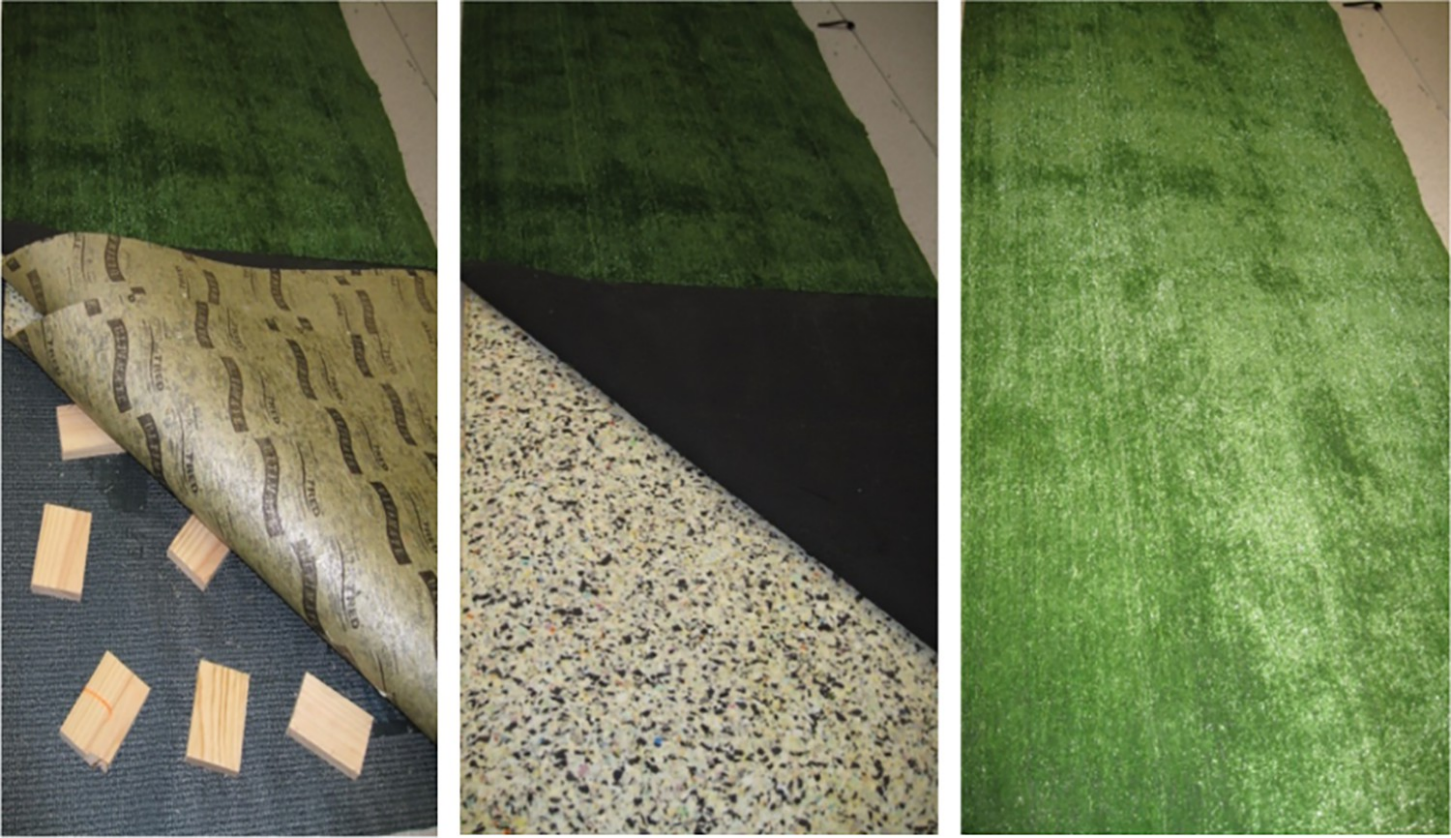
Uneven surface. This consisted of wooden blocks glued onto a mat (left) which were overlayed with foam (centre) and artificial grass (right).

There were two main walking conditions: regular walking and dual tasking. The dual tasks were serial subtraction tasks and verbal fluency tasks. There were four trials for each condition. The order of these walking conditions was randomized across participants. The participants were requested to walk three complete laps (six walking trials) and the first lap was taken as a warm-up trial and the second and third laps were used for analysis. Data from both lower limbs were used and the averages of the four trials were obtained for each variable. The even-surface walkway was flat, smooth, and free from obstacles. The uneven-surface walkway was constructed as per precedent [61] and consisted of randomly positioned small wooden blocks covered with layers of foam and artificial grass (Fig 2).

### Dual-tasks

Two separate dual tasks that involved working memory and attention [62,63], and which are known to involve the prefrontal cortex [64], were used during walking. Each of these tests has previously been added to walking to create a dual-task for assessing PD dance interventions [65].

The first cognitive task was an untimed verbal fluency test (DT_VERB_). The participant was required to name as many words as possible that begin with a nominated letter. Eight letters were randomly selected from within the *relatively easy* category [66,67] (B, R, L, S, T, P, C, and M). The same letters were used for all participants, and responses were recorded verbatim. A score of 1-point was awarded for each correct word. The total score was the sum of the correct responses across the eight trials, with higher scores indicating greater verbal fluency.

The second cognitive task was an untimed serial subtraction task (DT_SUBT_). A randomly generated whole number to three digits (e.g., 748) was given to the participant as a starting number [68]. The participant was required to subtract seven from the starting number, verbalise the result, and continue the process until no further subtractions could be made. One-point was awarded for each correct subtraction, with higher scores indicating greater working memory and attention.

For both dual-tasks, decrements in walking under dual-task conditions were also expressed as a percentage of single task performance, commonly referred to as the dual-task cost (DTC = [dual-task–single-task]/single-task * 100) [65]. Gait velocity, stride length, and double support percentage, which have been commonly used to measure gait-related changes in PD were rescored as DTC [4,65]. A higher DTC score indicates a greater cost of walking (worse gait).

### Intervention versus treatment as usual

The intervention, which was based on the DfPD^®^ approach [69], was led by DfPD^®^-trained instructors. The intervention commenced on 21^st^ Aug 2017 and continued for three months as a two-hourly twice weekly class. According to our systematic review [31], the majority of the studies (12 out of 27) provided classes twice a week. When considering the duration of a dance class, it ranged from 60 to 90 minutes in past studies and most of the studies (15 out of 27) had 60-minute classes. The duration of the intervention ranged from two to 104 weeks and among them, most studies (six) completed a twelve-week intervention. The feasibility of longer duration interventions needs to be carefully considered, given that Duncan (2012) had a 50% attrition rate over 12 months [70]. Health care and exercise professionals commonly use the Frequency, Intensity, Type, and Time (FITT) principle when prescribing exercises [71]. Based on the past literature we considered a FITT principle of one-hour dance class twice a week for twelve weeks for the current study. Classes started with a seated warm-up, followed by “barre” work (supported standing activities using the chairs), then moving across the floor, practicing a longer choreographic sequence, and closing with a warm-down activity [72].

Each class was followed by a social tea/coffee break. Participants could attend with a family member, friend, or caregiver. Each class had 3–4 volunteer assistants who were paired with participants who demonstrated poor balance. Each class started with a 30-minute seated dance, followed by 10–15 minutes each of standing dance with support, then standing dance movement across the floor. The intervention was progressively advanced over time by gradually introducing more challenging steps such as oppositional use of the arms and legs, multiple direction changes, the complexity of sequences, and increasing the number of repetitions and duration of activities. The intervention was added to each participant’s usual PD care.

Seated dance techniques included gentle upper body movement (10 minutes), slow and fast feet sequences (10 minutes), improvisational dances, and dance patterns which involved articulation of the spine and head, facial expressions, and vocalization (10 minutes). The classes also used dynamic and percussive movements and rhythmic patterns for both the upper and lower body within the other exercises. Standing dance contained repeated stretching of the leg and pointing the foot (‘‘tendu” a basic ballet step) (5 minutes), bending the knees with feet in various positions (plié), transfer of weight between feet, and extended arm patterns or port de bras (10 minutes) [69]; these movements were performed supported while standing (holding the back of a chair). The standing dance consisted of a mixture of choreographed sequences, locomotion patterns across the floor, improvisation, theatrical interpretation, and group dancing (15 minutes). These dance classes included aspects of ballet, modern dance, choreographic repertory, jazz, tap, Flamenco, and Scottish Dance. The dance classes were made progressive by increasing the number of repetitions and introducing more challenging dance patterns throughout the intervention period. Teaching methods incorporated verbal instruction, aesthetic imagery, attention to visual focus, repetition, rhythm, and imitation of movement [73]. Recorded music, which ranged from classical to modern, was carefully selected by the senior dance teacher.

The CG participants received their PD treatment as usual. This comprised routine clinical care for each patient. After baseline assessment, the research team made minimal contact with the participants in this group, apart from to schedule post-intervention assessments.

### Procedure

For each surface type, the spatiotemporal gait analysis was performed under three conditions (regular walking, DT_VERB_, DT_SUBT_). Altogether there were six tests for each occasion (2 surfaces X 3 conditions), the order of which was randomized across the participants. The six trials were performed on two occasions (baseline and post-intervention). Before each test, 41 retro-reflective markers were placed on the specific anatomic points of the subject’s body, according to the Vicon plug-in gait model [59]. To minimize errors, the same experienced investigator attached the markers, labelled, and analyzed the spatiotemporal data, and administered and scored the cognitive tests. For each trial, the participant was instructed to walk barefoot from the start to the end of the walkway at a self-selected pace. One practice trial for each condition was allowed before the tests commenced. For dual-task tests, the participant was requested to start walking while performing the cognitive task as soon as the starting number or letter was spoken to them.

### Statistical analysis

The independent-samples t-test was used to compare the means between two continuous, dependent variables (e.g., Age, Levodopa equivalent dose etc.). The Chi-Square test of independence was used to determine if there is a significant relationship between two nominal variables (Gender with groups and level of education with groups). However, chi-square test of independence demonstrates the violation of assumption where all cells should have expected counts greater than or equal to five which means there is not an adequate sample size to run the chi-square test of independence which can maximize the risk of making wrong decision. Therefore, the Fisher’s exact test findings was also used to confirm whether there is a significant association.

LMM) with factors of the group (dance, control) and condition (regular, dual-task-A, dual-task-B) was used. Separate analyses were performed for even and uneven surfaces. The dependent variable was a spatiotemporal gait parameter or its DTC derivative. Separate univariate analyses for each condition were used to compare the dance and control groups. Analyses were undertaken on the change (pre-post) scores. A preliminary analysis found that there were more females in the dance group (82.4%) than in the control group (37.5%; χ^2^ (1, N = 33) = 6.94, *p* = 0.008). Gender was therefore considered as a covariate in subsequent analyses. Although disease severity (MDS-UPDRS score) of the groups was statistically comparable at baseline (p>0.05), because there was large variability in these data (DG range: 36 to 116, CG range: 27 to 111) this variable was also included as a covariate. Effect sizes (d) were calculated using the Campbell Collaboration online calculator [74]. All statistical procedures were conducted using SPSS 25 and were evaluated using an alpha of 0.05.

## Results

### Demographic characteristics

Apart from there being more females in the dance group (82.4%, age 65.8±11.7 years, *n* = 17) than in the control group (37.5%, age 67.0±7.7 years, *n* = 16; *p* = 0.013), the groups had similar characteristics at baseline (Table 1). Participants were four to six years post PD diagnosis and they had had less than one fall in the past six-months (DG = 3 fallers, CG = 2 fallers). No adverse events (e.g. falls) were reported during the dance classes.

### Dual-tasks: Cognitive component

The performance and accuracy of the cognitive component of the dual-tasks were not statistically different for the groups during even and uneven surface trials at baseline and post-intervention (Table 2).

**Table 2:** Accuracy of the verbal fluency and serial subtraction tasks.

Surface	Time point	*Serial subtraction*						*Verbal fluency*					
		*Average total number of subtractions* ^*1*^		*Number of* *correct subtractions*		*Accuracy %*		*Total number of words* ^*2*^		*Number of* *correct words*		*Accuracy %*	
		***Dance*** ***(n = 17)***	***Control*** ***(n = 16)***	***Dance*** ***(n = 17)***	***Control*** ***(n = 16)***	***Dance*** ***(n = 17)***	***Control*** ***(n = 16)***	***Dance*** ***(n = 17)***	***Control*** ***(n = 16)***	***Dance*** ***(n = 17)***	***Control*** ***(n = 16)***	***Dance*** ***(n = 17)***	***Control*** ***(n = 16)***
**Even Surface**	Pre	4.50 (1.64)	5.18 (2.57)	4.20 (1.88)	4.66 (2.57)	91.25 (13.38)	87.42 (10.77)	6.51 (2.14)	5.46 (1.49)	6.26 (2.07)	5.39 (1.57)	96.30 (3.77)	98.08 (6.17)
	Post	5.06 (1.41)	4.89 (2.57)	4.58 (1.51)	4.50 (2.34)	89.33 (12.39)	92.03 (8.36)	7.44 (2.01)	6.14 (2.04)	7.37 (2.04)	5.91 (1.96)	98.45 (2.31)	96.29 (3.54)
		***Dance*** ***(n = 17)***	***Control*** ***(n = 14)***	***Dance*** ***(n = 17)***	***Control*** ***(n = 14)***	***Dance*** ***(n = 17)***	***Control*** ***(n = 14)***	***Dance*** ***(n = 17)***	***Control*** ***(n = 14)***	***Dance*** ***(n = 17)***	***Control*** ***(n = 14)***	***Dance*** ***(n = 17)***	***Control*** ***(n = 14)***
**Uneven surface**	Pre	5.10 (2.26)	5.50 (2.80)	4.52 (2.39)	4.89 (2.86)	85.76 (15.67)	86.04 (11.73)	6.69 (2.19)	5.41 (1.50)	6.35 (2.30)	5.27 (1.59)	94.39 (9.72)	96.87 (5.20)
	Post	5.73 (1.65)	5.67 (2.67)	5.23 (1.67)	5.14 (2.99)	90.74 (9.09)	85.94 (17.57)	8.30 (2.64)	6.70 (2.12)	8.17 (2.58)	6.39 (2.06)	98.42 (2.88)	95.60 (5.29)

^1^ Average number of total subtractions in a 6-meter walking trial. Random 3-digit numbers were used.

^2^ Average number of total words in a 6-meter walking trial. The letters used were “B, R, L, S, T, P, C, and M”. These letters were randomised across the walking conditions.

### Spatiotemporal gait analysis

#### Even surface

At baseline, there was no significant group difference for any spatiotemporal gait variable (Supplementary material: Annex I in S2 File). The DG improved significantly compared to the CG with and without a dual-task. The overall LMM analysis revealed that the DG had higher gait velocity (p = 0.017), cadence (p = 0.039), step length (p = 0.040), and stride length (p = 0.041) compared to the CG during regular walking (Supplementary material: Table 3, Supplementary material: Annex II-V in S3 File). Univariate analysis of pre-post change scores in each of the three conditions, found significant improvements for the DG compared to controls on four spatiotemporal measures in the DT_VERB_: velocity (p = 0.035), cadence (p = 0.034), step length (p = 0.039), with one exception; the group difference for step length approached but did not reach statistical significance (p = 0.053). The DG also exhibited significant improvement compared to the CG during DT_SUBT_ in the measures of gait velocity (p = 0.012), cadence (p = 0.021), step length (p = 0.018), and stride length (p = 0.151). There were no differences between the DG and CG for DTC change scores for any of the gait parameters on the even surface (Table 4).

**Table 3:** Pre-post change score of the spatiotemporal parameters of gait during even surface walking without a secondary task, and with cognitive secondary tasks.

Gait parameters	Regular walking Mean change score^2^ [95% CI]			Verbal fluency Mean change score^2^ [95% CI]			Serial subtraction Mean change score^2^ [95% CI]		
	Dance^1^ (n = 17)	Control^1^ (n = 16)	p-value^3^ Effect size^4^	Dance^1^ (n = 17)	Control^1^ (n = 16)	p-value^3^ Effect size^4^	Dance^1^ (n = 17)	Control^1^ (n = 16)	p-value^3^ Effect size^4^
Gait velocity (ms^-1^)	0.150 [0.075, 0.226]	0.013 [-0.056, 0.082]	**p = 0.017** d = 0.976	0.140 [0.061, 0.22]	0.015 [-0.056, 0.086]	**p = 0.035** d = 0.866	0.146 [0.069, 0.224]	-0.003 [-0.075, 0.068]	**p = 0.012** d = 1.033
Cadence (steps/sec.)	0.120 [0.053,0.188]	0.015 [-0.046,0.077]	**p = 0.039** d = 0.842	0.136 [0.067, 0.207]	0.024 [-0.041, 0.089]	**p = 0.034** d = 0.672	0.123 [0.053, 0.193]	0.0001 [-0.064, 0.065]	**p = 0.021** d = 0.905
Step length (metres)	0.044 [0.018,0.070]	0.004 [-0.020,0.027]	**p = 0.040** d = 0.809	0.041 [0.015, 0.068]	0.0001 [-0.024,0.025]	**p = 0.039** d = 0.828	0.048 [0.022, 0.075]	0.001 [-0.024,0.025]	**p = 0.018** d = 0.951
Stride length (metres)	0.088 [0.036,0.140]	0.008 [-0.039,0.055]	**p = 0.041** d = 0.843	0.083 [0.03, 0.137]	0.006 [-0.043, 0.055]	p = 0.053 d = 0.779	0.097 [0.044, 0.151]	-0.001 [-0.050, 0.048]	**p = 0.015** d = 0.992
Stance phase (%)	-0.745 [-1.574,0.084]	-0.144 [-0.905,0.618]	p = 0.324 d = 0.387	0.093 [-0.813, 0.999]	0.141 [0.707, 0.989]	p = 0.942 d = 0.029	0.498 [-1.399, 0.403]	0.176 [-0.669, 1.021]	p = 0.311 d = 0.187
Swing phase (%)	0.745 [-0.084,1.574]	0.144 [-0.618,0.905]	p = 0.324 d = 0.387	-0.093 [-0.999, 0.813]	-0.141 [-0.989, 0.707]	p = 0.942 d = 0.029	-0.498 [-0.403, 1.399]	-0.176 [-1.021, 0.669]	p = 0.311 d = 0.187
Double support (%)	-1.490 [-3.147,0.168]	-0.288 [-1.811,1.235]	p = 0.324 d = 0.387	0.186 [-1.626, 1.997]	0.282 [-1.415, 1.979]	p = 0.942 d = 0.029	0.996 [-2.798, 0.807]	0.352 [-1.338, 2.041]	p = 0.311 d = 0.187
Single support (%)	1.490 [-0.168,3.147]	0.288 [-1.235,1.811]	p = 0.324 d = 0.387	-0.186 [-1.997, 1.626]	-0.282 [-1.979, 1.415]	p = 0.942 d = 0.029	-0.996 [-0.807, 2.798]	-0.352 [-2.041, 1.338]	p = 0.311 d = 0.187
Stride time variability	-13.24 [-24.08, -2.40]	-0.34 [-7.11, 6.43]	p = 0.249 d = 0.743	-21.19 [-46.58, 4.21]	-8.13 [-24.22, 7.96]	p = 0.272 d = 0.029	-18.50 [-42.89, 5.89]	-1.04 [-12.75, 10.67]	p = 0.318 d = 0.470

Notes. n = 23, Dance and control group were not significantly different for any measure at baseline, ^2^ Linear Mix Model applied for change scores. Covariates: gender and disease severity. ^3^ Bold values are significant (*p*-value < 0.05), ^4^Effect sizes (*d*) calculated using Campbell Collaboration online calculator.

**Table 4:** Calculation of dual task cost for gait variables during verbal fluency task and serial subtraction tasks on even surface and uneven surface.

*Gait parameters*	*Walking surfaces*	*Conditions*	*DTC Pre-test* ^*1*^ *Mean (SD)—%*		*DTC Post-test* *Mean (SD)—%*		*Change scores Mean*^*2*^ *- %*^*2*^ *[95% CI]*		*p-value* ^*3*^	*Effect size* ^*4*^
			*Dance* *(n = 17)*	*Control* *(n = 16)*	*Dance* *(n = 17)*	*Control* *(n = 16)*	*Dance* *(n = 17)*	*Control* *(n = 16)*		
**Gait velocity**	Even surface	Serial subtraction	-22.74 (13.96)	-13.43 (10.73)	-20.14 (16.79)	-15.33 (12.04)	2.60 [-16.41, 27.27]	-1.90 [-22.91, 22.40]	0.56	0.37
		Verbal fluency	-20.19 (13.57)	-15.12 (12.26)	-17.73 (17.29)	-15.55 (12.96)	2.45 [-30.55, 18.41]	-0.42 [-23.18, 30.22]	0.33	0.22
	Uneven surface	Serial subtraction	-26.34 (14.97)	-12.36 (7.34)	-17.99 (15.81)	-13.28 (8.07)	8.35 [-13.40, 54.77]	-0.91 [-12.75, 14.32]	**0.05**	0.68
		Verbal fluency	-20.36 (13.58)	-16.82 (10.56)	-15.14 (15.61)	-16.59 (11.24)	5.22 [-11.65, 34.62]	0.23 [-12.80, 15.87]	0.15	0.51
**Stride length**	Even surface	Serial subtraction	-13.96 (12.31)	-7.22 (6.00)	-11.94 (10.78)	-8.29 (9.18)	2.01 (-9.26, 23.22)	-1.06 (-17.24, 13.25)	0.92	0.39
		Verbal fluency	-11.68 (10.82)	-8.19 (7.59)	-10.38 (12.20)	-8.89 (8.46)	1.30 (-14.75, 17.57)	-0.70 (-19.01, 18.76)	0.42	0.24
	Uneven surface	Serial subtraction	-13.83 (13.56)	-5.50 (6.64)	13.83 (13.56)	-5.50 (6.64)	4.03 (-19.14, 27.60)	-1.64 (-10.02, 15.13)	**0.05**	0.44
		Verbal fluency	-9.83 (11.33)	-8.94 (7.06)	-8.69 (11.19)	-10.87 (7.95)	1.14 (-7.96, 10.69)	-1.93 (11.78, 12.32)	0.16	057
**Double support %**	Even surface	Serial subtraction	14.37 (14.14)	10.16 (12.05)	18.27 (13.89)	12.68 (7.80)	3.91 [-41.19, 29.24]	2.52 [-8.63, 26.35]	0.94	0.09
		Verbal fluency	11.61 (11.78)	11.35 (12.45)	19.95 (18.49)	13.48 (12.17)	8.33 [-23.95, 40.55]	2.49 [-17.96, 24.12]	0.31	0.41
	Uneven surface	Serial subtraction	24.10 (23.36)	6.71 (10.75)	4.01 (20.27)	8.42 (19.18)	-20.09 [-57.78, 24.83]	1.70 [-38.20, 43.35]	**0.01**	0.98
		Verbal fluency	18.85 (19.76)	13.83 (15.24)	5.43 (16.86)	12.40 (18.10)	-13.41 [-70.76, 18.29]	-1.43 [-65.73, 23.26]	0.25	0.55

Notes. n = 23, ^1^DTC (Dual Task Cost) = [dual-task–single-task]/single-task * 100) ^2^Change scores are adjusted for gender and disease severity (UPDRS) ^3^General linear model applied for change scores. Bold values are significant (*p*-value < 0.05). ^4^Effect sizes (*d*) calculated using Campbell Collaboration online calculator.

#### Uneven surface

At baseline, there was no significant group difference for any spatiotemporal gait variable for the uneven surface (Supplementary material: Annex I in S3 File). The overall LMM analysis exhibited no significant group main effect for any of the gait parameters. However, there was a significant main effect for the task and a significant group*task interaction for stance phase (%), swing phase (%), double support (%), and single support (%) (Table 5, Supplementary material: Annex VI-VIII in S3 File). The univariate analyses of pre-post change scores for regular uneven surface walking found that the DG had a longer double support phase and stance phase compared to controls. While DT_VERB_ condition had no significant group differences for any gait parameter, in the DT_SUBT_ condition, the DG improved significantly compared to the controls on gait velocity (p = 0.048), cadence (p = 0.026), and step length (p = 0.051). On the uneven surface there was DTC improvement in the DG relative to controls for three gait parameters from DT_SUBT;_ gait velocity (p = 0.05), stride length (p = 0.05), and double support (p = 0.01) (Table 4).

**Table 5:** Pre post change score of the spatiotemporal parameters of gait during uneven surface walking without a secondary task, and with cognitive secondary tasks.

*Gait parameters*	*Regular walking* *Mean change score*^*2*^ *[95% CI]*			*Verbal fluency* *Mean change score*^*2*^ *[95% CI]*			*Serial subtraction* *Mean change score*^*2*^ *[95% CI]*		
	*Dance* ^*1*^ *(n = 17)*	*Control* ^*1*^ *(n = 16)*	*p-value*^*3*^ *Effect size*^*4*^	*Dance* ^*1*^ *(n = 17)*	*Control* ^*1*^ *(n = 16)*	*p-value*^*3*^ *Effect size*^*4*^	*Dance* ^*1*^ *(n = 17)*	*Control* ^*1*^ *(n = 16)*	*p-value*^*3*^ *Effect size*^*4*^
Gait velocity (ms^-1^)	0.130 [0.046,0.214]	0.040 [-0.039,0.12]	p = 0.162 d = 0.570	0.138 [0.054, 0.222]	0.046 [-0.034, 0.126]	p = 0.152 d = 0.583	0.165 [0.081, 0.249]	0.036 [-0.044, 0.116]	**p = 0.048** d = 0.817
Cadence (steps/sec.)	0.096 [0.011,0.181]	0.010 [-0.071,0.092]	p = 0.186 d = 0.531	0.152 [0.067, 0.237]	0.042 [-0.040, 0.124]	p = 0.092 d = 0.672	0.170 [0.085, 0.255]	0.022 [-0.060, 0.104]	**p = 0.026** d = 0.905
Step length (metres)	0.045 [0.012,0.078]	0.020 [-0.012,0.051]	p = 0.307 d = 0.396	0.046 [0.013, 0.079]	0.011 [-0.021, 0.043]	p = 0.174 d = 0.555	0.069 [0.036, 0.102]	0.019 [-0.013,0.051]	**p = 0.051** d = 0.793
Stride length (metres)	0.091 [0.028,0.155]	0.050 [-0.01,0.111]	p = 0.392 d = 0.347	0.092 [0.029, 0.155]	0.027 [-0.034, 0.088]	p = 0.182 d = 0.540	0.132 [0.069, 0.195]	0.038 [-0.023, 0.099]	p = 0.057 d = 0.781
Stance phase (%)	0.843 [-0.372,2.059]	-1.002 [-2.189,0.185]	**p = 0.049** d = 0.067	-1.076 [-2.292, 0.139]	-1.194 [-2.397, 0.009]	p = 0.899 d = 0.050	-2.072 [-3.295, -0.848]	-0.925 [-2.128, 0.279]	p = 0.221 d = 0.480
Swing phase (%)	-0.843 [-2.059,0.372]	1.076 [-0.139,2.292]	**p = 0.049** d = 0.067	1.076 [-0.139, 2.292]	1.194 [-0.009, 2.397]	p = 0.899 d = 0.050	2.072 [0.848, 3.295]	0.925 [-0.279, 2.128]	p = 0.221 d = 0.480
Double support (%)	1.687 [-0.744,4.118]	-2.003 [-4.377,0.37]	**p = 0.049** d = 0.417	-2.152 [-4.584, 0.279]	-2.388 [-4.795, 0.019]	p = 0.899 d = 0.049	-4.143 [-6.590, -1.696]	-1.849 [-4.256, 0.558]	p = 0.221 d = 0.537
Single support (%)	-1.687 [-4.118,0.744]	2.003 [-0.37,4.377]	**p = 0.049** d = 0.417	2.152 [-0.279, -0.019]	2.388 [-0.019, 4.795]	p = 0.899 d = 0.049	4.413 [1.696, 6.590]	1.849 [-0.558, 4.256]	p = 0.221 d = 0.537
Stride time variability	-13.47 [-57.95, 31.00]	-7.82 [-25.37, 9373]	p = 0.434 d = 0.085	-53.64 [-105.05, -2.23]	10.99 [-40.96, 62.95]	p = 0.286 d = 0.666	63.19 [-102.82, -23.56]	-12.59 [-47.13, 21.94]	p = 0.399 d = 0.719

Notes. n = 23, ^1^ Dance and control group were not significantly different for any measure at baseline, ^2^ Linear Mix Model applied for change scores. Covariates: gender and disease severity. ^3^ Bold values are significant (*p*-value < 0.05), ^4^Effect sizes (d) calculated using Campbell Collaboration online calculator.

## Discussion

This study investigated the effects of gait spatiotemporal parameters of a dance intervention for people with PD compared to a PD control group who did not dance (treatment as usual). At baseline and post-intervention, gait was assessed on even and uneven surfaces while walking regularly, and while engaging with one of two different cognitive tasks. The intervention was based on a widely used model that has shown promise in a range of gait and non-gait outcomes. We aimed to determine if the DfPD^®^ gait benefits might extend to everyday conditions including walking on different surfaces and walking while dual tasking, which are novel additions to a gait evaluation for PD dance interventions.

This study supported the hypothesised benefit of the DfPD^®^ program on a wide range of spatiotemporal gait measures and under novel conditions. The largest and most consistent benefit was observed for even surface walking, including while dual-tasking. There were mixed results for uneven surface walking, but importantly some benefits were found. At baseline, the gait parameters (velocity, cadence, stride length, and double support %) for the DG were similar to previously reported values for PD fallers (15). Following the intervention, these parameters decreased to a low risk for falls (15). This suggests that the DfPD^®^ approach can benefit gait on a wider range of measures than previously demonstrated and that the benefits are clinically significant.

### Even surface walking

The DfPD^®^ intervention significantly improved gait velocity, cadence, and step length during regular even surface walking. This is consistent with the benefits reported in previous studies using functional mobility tests [14,33,38,39,41,42,45,75,76]. The present study is the first evaluation of the DfPD^®^ approach using spatiotemporal gait parameters. When compared to other PD dance programs with similar measures, most of which used gait velocity only [13,19,33,38,40,43,44,77], our findings are consistent with two studies [19,38] while three studies showed no change [13,33,43]. Importantly, the improvement in gait velocity in our study was also clinically significant [78]. The minimally clinically important difference (MCID) for gait speed among medicated people with PD ranged from 0.05 m/s to 0.22 m/s (by distribution-based analysis) [78] and in this study the improvement of gait speed (0.150 m/s, 95% confidence interval = 0.075–0.226) was within that range. We also found benefits on other spatiotemporal parameters [i.e. cadence (steps/min), stride length (m), and swing, single and double support (time or % per gait cycle] that have not improved in prior group comparisons (i.e. partnered vs non-partnered Tango or Tango vs Ballroom) [79,80]. Whilst the reasons for these discrepancies are a matter of speculation, this could be because we employed a treatment-as-usual control group rather than comparing dance styles (potentially diluting effects), or it could be because of our program. In single-sample studies, our findings for cadence and single support time are consistent with the literature, including the findings for dance programs of adapted Argentine Tango [80,81] and contact improvisation [45,79]. It seems that dance interventions in general can improve spatiotemporal gait parameters for even surface regular walking, and this study provides the first, thorough demonstration of these benefits using the DfPD^®^ approach.

Following the dance intervention, there was a significant improvement in gait velocity, cadence, and step length when each cognitive task was added to walking on the even surface. The improvement in gait velocity was found for both dual tasks (verbal fluency: 0.14 ms^-1^, serial subtraction: 0.15 ms^-1^) and exceeded the threshold for an MCID. Compared to the mixed results from the previous dance intervention dual-task gait studies [13,33,36–39], our findings are consistent with studies that used verbal fluency [38] but not serial subtraction [82,83]. Dual-task cost for even surface walking was consistent with previous research [65] and was similar for both these dance and control groups. Taken together the results reinforce the notion that a DfPD^®^-style intervention could have wider benefits than previously identified, including when dual-tasking.

### Uneven surface walking

This study did not find the hypothesised improvements in spatiotemporal gait parameters for regular uneven surface walking. Compared to controls we found that the DG had significantly increased stance and double support phases and a corresponding decrease in swing phase; characteristics that are synonymous with a more cautious gait. The changes in gait when attempting to increase double support percentage trended toward changes when decreasing gait speed. Uneven surface trials have not previously been studied in PD dance interventions. However, it could be that as a result of the program, the DG participants took greater care walking in the higher-risk environment, which could be interpreted as an improvement. This test should be replicated in future research given that everyday walking environments are comprised of different irregular surfaces [84] and such characteristics cause increased fatigue, fear of falling, and more frequent freezing episodes in people with PD [85].

The dance intervention resulted in improvements in gait velocity, cadence, and step length while walking on an uneven surface and performing serial subtractions. There was also a significant improvement in dual-task cost for the dance group for the subtraction condition. However, no gait improvements were observed for DT_VERB_. This illustrates the importance of evaluating gait performance on more challenging surfaces which mimic everyday environments. It also lends support to the DfPD^®^ approach as an effective method for improving dual-task performance, which has previously proven resistant to modification [65]. It is unclear why this benefit was observed for the subtraction task only. However, it has been reported that in healthy individuals a verbal fluency task did not show any effect on stride velocity whereas an arithmetic task instigated a decline in gait speed and the ability to enumerate numbers compared to single-task conditions [86]. It is plausible that the subtraction task could have demanded more attention and executive function along with the additional physical challenge provided by the uneven surface. Adapting locomotor movements to varied terrains is important in daily life, and the current study provides support for future research that improves gait under different tasks and environmental constraints.

### Why did gait improve?

The DfPD^®^ classes possess unique features that assist this model to stand out amidst other different dance styles. The DfPD^®^ methodology is not just about the mix of styles included but how the intervention is delivered to participants; with an artistic sensibility that integrates aesthetic imagery, musicality, and aesthetic goal-setting as integral to the dance experience [48]. All DfPD^®^ classes incorporate specific components that are considered essential to the classes’ success. Firstly, the classical and contemporary technique training is taught through a progressive warm-up which may contribute to building strength, flexibility, and coordination skills. Secondly, the improvisation, co-creation, and aesthetic interpretation stimulate creativity and the imagination of the participants. Thirdly, the choreographic repertory and new movement sequences help participants to develop cognitive strategies. In addition, the circle dances, line dances, and scene work foster social interaction and create a sense of connection and community. There is also a strong musicality which informs every aspect of the class so that melody, structure, and rhythm guide and inspire participants’ physical and emotional exploration and expression [72].

Gait improvement following our intervention may be because the program attributes promoted different attentional cues for movement (visual, auditory, somatosensory). Cueing strategies have demonstrated improved gait in people with PD [21–25]. External cues have been hypothesised to bypass defective basal ganglia using alternative pathways [26]. The music that is used in dance is thought to provide an important auditory cue to move [22] via variations in rhythm and tempo. Tactile feedback could also cue movement; for example when holding hands with a dance partner [69]. Rhythmic auditory cues and attentional strategies have been effective in improving walking speed and step amplitude when dual-tasking [21]. Gait may have improved in this study because the intervention: promoted generalized use of external cues, developed movement skills or confidence, or was spurred by other program attributes.

The gait benefits seen in dual-tasking warrant close consideration. Since dual tasking relies on executive function and the ability to divide attention, the improvement may have been due to the cognitive skills practised in dancing, such as learning and remembering dance sequences [10]. During dancing, participants must rapidly and skilfully switch between dance routines, processes which involve executive control and working memory [87]. The “dancer” must attend to the dance teacher’s instruction and performance, plan and execute the required movement, and maintain control and balance throughout the artistic performance. These task-specific aspects of multitasking may thus be related to improved dual-task gait resulting from participation in the DfPD^®^ classes.

The mechanism of improvement in gait and dual task gait could be due to improvement in multiple functional measures such as balance, muscle strength, cognition, disease severity, and fine manual dexterity. Our assessment included some of these measures and the findings have been published previously [88,89]. The dance intervention demonstrated significant improvement in selected cognitive skills (executive function and episodic memory) measured using the National Institute of Health toolbox® (NIH Toolbox®), balance measured using the Berg Balance scale, Tinetti balance and gait assessment, and Mini Best Test. Improvements were also noted in disease severity and among the four sub-scales of MDS-UPDRS, the MDS-UPDRS-III, which is an assessment of motor skills (gait, balance, bradykinesia, and rigidity), had the largest effect size (d = 2.01). Therefore, we speculate that the improvement in gait could be related to the changes in the above functional measures.

### Feasibility

While the pilot research project conducted in Queensland Ballet, based on the DfPD^®^ programme concluded that the dance classes influenced people living with PD in numerous ways, the study was limited in the experimental design, sample size, and lack of objective assessments. The current study used objective and detailed assessment of gait, dual tasking, cognition (using the NIH-toolbox), as well as real-time activity measures for habitual physical activity (using ActivPAL accelerometers), and some of these findings are discussed in other papers [88,89]. While the current paper presents only the spatiotemporal parameters of gait, the kinematic data captured using 3D motion capture are yet to be published. Using different surfaces and different cognitive tasks also becomes novel in this study. Also, this study provides information for implementing a large-scale RCT by confirming the feasibility of assessing multiple domains.

The class participants did not experience any adverse events such as falls during the class. Classes were designed to facilitate participants at different levels of capabilities. The seated dance provided them with a confident start and standing with chairs for support allowed them a safe transition. The progressive standing dance was for those who felt safe and confident, while others were given the choice to continue as seated dancers. Safety and confidence were further reinforced using volunteers who were able to pair with participants who demonstrated poor balance. Overall, the participants had a safe and comfortable class experience. The dance classes promoted the social network and fostered interpersonal relationships. Given the low attrition rate, it appears the programme was acceptable to most participants. Following the completion of the study, the participants requested an ongoing DfPD^®^ class. This confirms DfPD^®^ model has high demand as a therapeutic intervention among the intended population.

### Limitations

The study was limited by the sample size. We acknowledge that the small sample size reduces the power of the study and increases the margin of error. This was in part due to feasibility constraints on the pilot study class size. Additional parallel groups should be offered in future research, such as a group with Argentine Tango. We speculate that the benefits observed in this research could be due to the complex, multi-dance style design of the intervention we used; however, this idea should be tested further. Group allocation was pseudo-random as it was affected by participants’ availability among other things. This resulted in an unequal male-to-female ratio in the two groups and although this was controlled for during statistical analyses, this is a limitation of this study. Insight into the mechanisms for change in the functional measures is challenging and could be due to multiple factors such as balance, cognition, and muscle strength. While the improvements in balance and cognition have been reported in papers published previously, not including a measure of muscular strength is a limitation. The current study only presents the spatiotemporal variables, and we acknowledge that the gait analysis can extend well beyond spatiotemporal variables, to kinematic and kinetic parameters. In addition, more surface variations could be provided such as steps and inclines to assess how the benefits might extend to other everyday conditions.

## Conclusion

This study indicates the very high potential that a large-scale RCT will also find positive and clinically meaningful benefits for dance on spatiotemporal even surface gait parameters with and without a dual-task and uneven surface walking with a serial subtraction task. It also demonstrates the practicality of safely undertaking a large-scale randomised controlled trial of this type of program. Future studies should examine the durability of these benefits, including transferability to home environments and community ambulation, and if they extend to reducing falls risk.

## Supporting information

S1 Checklist TREND statement checklist. (DOC)

S1 Dataset (XLSX)

S1 File (DOCX)

S2 File (DOCX)
