# Supplementary material for: Effects of dance on gait and dual-task gait in Parkinson’s disease
Source: PLoS One. 2023 Jan 24;18(1):e0280635. doi: 10.1371/journal.pone.0280635 (PMC9873168; doi:10.1371/journal.pone.0280635)
Supplement: S1 File — (DOCX) [file pone.0280635.s003.docx]

University Human Research Ethics Committee (UHREC)

**APPLICATION FOR REVIEW OF NEGLIGIBLE / LOW RISK RESEARCH INVOLVING HUMAN PARTICIPANTS**

***NOTE*** • *All answers should be written* ***in simple and non-technical language*** *that can be* ***easily understood by the lay reader****.*

- *You must provide an answer to each question –* ***N/A is not acceptable****.*

**SECTION A: RESEARCH PROPOSAL OVERVIEW**

**A1 Summary Information**

**A1.1 Project title** (200 character limit including spaces)

Effects of Dance for Parkinson’s on gait and cognitive skills in Parkinson’s Disease

# A1.2 Brief project summary in LAY LANGUAGE

(i.e. in plain English and ensure when using acronyms you spell them out in the first instance)

The Dance for Parkinson's classes at Queensland Ballet (QB) are modelled on the Dance for PD® programme (DfPD®) which was initiated in New York by the Brooklyn Parkinson Group in collaboration with the Mark Morris Dance Group in 2001 (Westheimer, 2008). These classes are offered in partnership by Dance for Parkinson's Australia (DPA) with Queensland Ballet, modeled on the DfPD® program. The QB Dance for Parkinson's (DfP) classes comprise of 75 minute dance classes with live music accompaniment, and employ strategic methods such as cuing, imagery, repetition, variation and imitation of discrete parts of dance sequences to gain superior control of posture, balance, and movement (Hackney & Earhart, 2009).

Although the effects of dance on people with Parkinson’s disease (PD) is progressively being investigated, there is limited literature with regard to the DfPD® programme. These recent studies do not provide definitive evidence, particularly with relation to gait and cognition. Therefore, the current project aims to determine the effectiveness of DfP on gait and cognitive skills in PD. This study will be conducted in two main sections as follows:

# Section one

The first section involves a pilot study, which aims to characterise the amount of activity (total limb acceleration) undertaken by people with PD during a DfP class at QB. Participants for this section will be recruited from the ongoing DfP class at QB and the study will be conducted at QB dance studios. Fifteen participants who are currently undergoing DfP at QB who fulfil the inclusion criteria will be recruited. Specific devices (ActivPALs) will be used to measure the posture and motion during everyday activities. This section of the study will be conducted at a single class and it will be a representation of all DfP classes. This representative class will be decided having discussed with DfP teachers. In addition, the participants will be requested to wear the device at home for five consecutive days to monitor their routine activities at home. These devices are planned to be used in the second section of the study. Gait, balance and cognitive skills will be measured using valid and reliable measuring tools and the assessment will be done one hour before starting the class at QB on the same day.

# Section two

The second section of the study aims at exploring the effectiveness of the DfP program on PD, primarily on gait and cognition. The balance, coordination, quality of life and caregiver burden will be measured as the secondary outcomes. This section of the study is planned to be conducted at QUT. The QUT DfP class will be modelled on the DfPD® programme and will be led by two DfPD® trained instructors who are routinely conducting DfP classes at QB. Having screened for eligibility, the participants will be randomly allocated to the experimental group and the control group. There will be thirty participants for each group. The allocation will be concealed from the investigators (investigator blinded). Experimental group will participate in DfP sessions twice a week for twelve weeks, while control group will follow their usual medication and exercises regime. Pre-test will be performed one week before the start of DfP classes while post-test will be performed one week after the last DfP class. A follow-up evaluation will be conducted during the 12th week after the last class. Valid and reliable measures will be used for measuring the primary and secondary outcomes (refer A 1.3 for details).

# A1.3 Provide an overview of your research participants and their involvement (max 250 words)

The purpose of this question is to gain a sense of who the participants will be, and what you expect them to do within the research

**Section one**

Participants for the pilot study will be recruited from the ongoing DfP classes at QB. Fifteen eligible participants who are diagnosed as having idiopathic PD, within the age range of 30-85 years and who fulfil the inclusion criteria will be recruited. Participants will be screened for eligibility using Hoehn and Yahr (H&Y) disability scale (Goetz et al., 2004), Movement Disorder Society Unified Parkinson's Disease Rating Scale (MDS-UPDRS) (Goetz et al., 2007) and Addenbrooke’s Cognitive Examination (ACE) (Reyes et al., 2009).

- Activity monitoring during the class: The ActivPAL (PAL Technologies Ltd, Glasgow, UK), are small lightweight tri-axial accelerometers which use algorithms to record time spent sitting/lying, standing, and walking,

transitions and step count. These devices are worn on one upper limb and one lower limb and have the dimensions 4 cm X 2 cm X 0.5 cm and weigh 15 g. The ActivPALs also measures acceleration in three different anatomical planes (X, Y, and Z) which allow for the calculation of total acceleration (Aminian & Hinckson, 2012). These devices have a substantial processing capacity and memory allowing for continuous recording for periods of up to 10 days (Chastin et al., 2010). The participants will be requested to wear the devices during a class, while the whole class will be video recorded with an elapsed time indicator. It will assist in matching the limb acceleration with the particular activity the participant was performing. For videoing the class, informed written consent will be obtained from all the DfP class participants, those who participate in the study as well as not participating in the study. The image release consent form is attached herewith. In addition, participants will be requested to wear the device at home for five consecutive days in order to monitor their activity at home.

Measurement of gait, balance and cognition will be done one hour before starting the DfP class at QB.

- - Gait: Gait analysis will be conducted using GAITrite mat that is approximately nine metres long. Participants will perform three trials of comfortable forward walking and fast as possible forward walking. Spatiotemporal parameters of gait (i.e. step length, velocity) will be measured. The Freezing of Gait Questionnaire (FOG-Q) will be used as an assessment of freezing of gait severity (Giladi et al., 2009).
  - Balance: The mini-BESTest, Tinetti Mobility Test (TMT) and Activities-specific Balance Confidence (ABC) Scale will be used to measure the balance.
  - Cognitive skills: Montreal Cognitive Assessment (MoCA) will be used to measure participants’ cognitive skills (Gill, Freshman, Blender, & Ravina, 2008).

# Section two

This project has been designed as a single blinded parallel group randomized control clinical trial, which is planned to be conducted at QUT. Participants who are diagnosed as having idiopathic PD, in the age range of 30-85 years, who are able and willing to commit to attending dance classes twice a week for twelve weeks, will be recruited through distributing recruitment flyers to the pre-existing DfP class at QB, local advertisements and through contact with local PD support groups. Participants will be screened for eligibility using Hoehn and Yahr (H&Y) disability scale (Goetz et al., 2004), Movement Disorder Society Unified Parkinson's Disease Rating Scale (MDS-UPDRS) (Goetz et al., 2007) and Addenbrooke’s Cognitive Examination (ACE) (Reyes et al., 2009). The selected Participants need to be H&Y stages I-III in order to ensure they are in mild to moderate stage of the disease and more than 82 in ACE to exclude cognitive impairment.

Having been screened for eligibility, the participants will be randomly allocated to the experimental group and the control group, comprising of thirty participants in each group. The allocation will be concealed from the investigators (investigator blinded). The experimental group will carry out DfP sessions twice a week for twelve weeks, while the control group will lead their usual medication and exercises regime. Consented care-givers of the same participants will also be recruited.

Assessment protocol for the study is as follows:

- - Descriptive: Personal details including year of diagnosis with PD, other existing health and medical conditions, history and frequency of falls in the past six months, and current medication use will be recorded. All participants will remain anonymous by using a coding system for patient identities. The privacy and security of their medical records will be ensured.

To address the aims of this project, the following measures will be used at pre-test, upon completion of the dance classes and at follow up evaluation. Please see appendix one for details on the tools of measurement.

- - Gait: Gait analysis will be conducted using 11-camera Vicon data capturing system (VDCS) and GAITrite mat. The VDCS records the subjects while walking barefoot on a firm surface and an uneven surface pathway that is approximately nine metres long, with reflective markers attached to certain parts of the body. Participants will perform three trials of comfortable forward walking, fast as possible forward walking and dual task walking. During dual task walking, the participant will be engaged in dual tasks that involve executive function (e.g. serial subtraction task: counting down from a random number by sevens or verbal fluency task: naming as many words as possible that begins with a certain letter). Spatiotemporal parameters of gait, upper body movements and joint kinematics will be measured. The main focus of attention will be gait speed, stride length and step width.
  - Cognitive skills: Detailed cognitive assessment will be performed using the National Institutes of Health tool box (NIH Toolbox®). This tool has been validated for use in diverse cultures, ethnic and geographic groups, ages (30-85 years) and study types. This will be administered using an iPad. Selected tests in the cognitive battery will be used to assess the components of executive function (Weintraub, Dikmen et al. 2013). In addition, Montreal Cognitive Assessment (MoCA), which is a brief 30-point screening instrument that was

# A1.4a

developed and validated to identify patients with mild cognitive impairment will be used. The MoCA includes tests of the cognitive domains of executive and visuospatial function, memory, language, and attention (Gill et al., 2008).

- Balance: The mini-BESTest, Tinetti Mobility Test (TMT) and Activities-specific Balance Confidence (ABC) Scale will be used. The mini-BESTest is a 14-item test of dynamic balance (Leddy, Crowner, & Earhart, 2011). Tinetti Tool is a 16 item, 28-point tool which assesses balance and gait, and will take about 8-10 minutes to complete (Kegelmeyer, Kloos, Thomas, & Kostyk, 2007). ABC is an 11 point scale and ratings consist of from 0-100 for each item. This can be self-administered (Ashburn et al., 2014).
- Coordination: Upper limb coordination will be measured using the Nine-hole peg test (9HPT), which has demonstrated high test-retest reliability in people with PD (Earhart, 2011). It is anticipated that this testing will take approximately 5 minutes to complete.
- Activity monitoring: Participants will be requested to wear ActivPALs to monitor their activity at home and during the class. Firstly, each participant will have one ActivPAL attached to their mid anterior thigh and wear them for five days before the initiation of classes and another five days after completing the series of classes, in order to monitor their level of activity at home. Secondly, they will be required to wear the devices during the class to characterise the amount of activity (total limb acceleration) undertaken during the class. This will be done specifically during their first class and last class. During the class, ActivPALs will be worn one on the upper arm and the mid anterior thigh. Further information regarding the wearability of ActivPALs is provided in the information sheet for the PD participants. The entire class will be video recorded as previously mentioned in section one and the image release consent form is attached herewith.
- Quality of Life (QoL): The QoL of the PD participants will be assessed through the Parkinson’s Disease Questionnaire-39 (PDQ-39), which comprises 39 questions, each of them with five different answer options (never, occasionally, sometimes, often or always) (Filippin, Lobo da Costa, & Mattioli, 2010).
- Caregiver burden: The Zarit Burden Interview (ZBI) will be used to determine the level of caregiver strain. In addition they will be asked to participate in semi-structured feedback interviews as mentioned below.

Qualitative evaluation:

- Participants will be asked to participate in semi-structured feedback interviews identifying whether the dance classes met their expectations, what (if anything) they feel they have gained from participating, and what motivated them to continue attending. Interviews will last approximately 45 minutes to 1 hour and will be either conducted at QUT, over the phone or via Skype, according to the participant’s convenience at a post time point (on completion of the series of DfP classes).
- Caregivers will also be invited to participate in semi-structured feedback interviews in order to determine the impact the dance class had on them and the PD participant. Interviews will last approximately 45 minutes to 1 hour and will be either conducted at QUT, over the phone or via Skype, according to the caregiver’s convenience. This will also be done at a post time point (on completion of the series of DfP classes).

**Provide a summary of the merits of this proposed research (in LAY LANGUAGE) including the aims / hypotheses / research questions** (refer to Section 1 of the National Statement, NS1.1, when preparing your response)**.**

- Include potential contributions to the body of knowledge and methodological rigor (max **250** words).Briefly provide evidence that the proposed research is based on knowledge of the relevant literature, and provide a list of key references.You may also attach a research plan / methodology which does not substitute for the summary above – this attachment should be no longer than **6** pages.**NOTE:** Unless proposed research has merit (and the researchers who are to carry out the research have integrity) the involvement of human participants in the research cannot be ethically justified.

Although there are several studies investigating the effect of dance on people with PD, the studies are limited and do not provide definitive evidence in relation to effect of dance on gait and cognition. Specifically, the effectiveness on dual task walking has not been systematically evaluated in any of the previous studies. Notably, very few studies have undertaken a follow-up evaluation over a significant period of time. In fact, in the area of gait and cognition, very little conclusive evidence is available concerning the short and/or long term effects of DfPD programme. Therefore, this research argues that the DfPD programme has the potential to become an effective supportive approach for PD that could be valuable in the management of motor and cognitive symptoms, thereby contributing to the evidence base for improving dual tasking in PD, which has not been investigated in past studies related to DfPD© programme. It is anticipated that the findings will reveal new insights into the DfPD programme as a viable approach in the management of PD and, this would provide possible new solutions for clinicians and therapists who are engaged in the process of rehabilitating PD patients.

**Research context**

1. It is evident that impaired gait is a major cause of disability in people with PD and dance interventions have been argued to be effective in improving spatial as well as temporal parameters of gait (Duncan & Earhart, 2012). The quality of human gait pattern depends on an array of biomechanical features and therefore, to

# A1.4b

evaluate the quality, a detailed gait analysis is required including assessment of upper body motion and joint kinematics. Nevertheless, such a detailed gait analysis has not been done particularly regarding the DfPD programme.

1. Some evidence suggests that people with PD are more affected by dual-tasks than healthy older adults (Foley, Kaschel, & Sala, 2013). Earhart has argued that dance practice promotes the activation of regions that normally display low activity in PD (Earhart, 2009). Thus it could have implications in enhancing dual tasking in PD. This research project will be the first study to analyse spatiotemporal parameters of gait during dual task walking in relation to a DfPD programme.
2. Executive dysfunction (EF) is one major domain of cognitive deficits in PD, and to date, the effectiveness of DfPD programme on executive function has not been investigated (Rios Romenets, Anang, Fereshtehnejad, Pelletier, & Postuma, 2015).
3. Numerous studies have used accelerometers to objectively quantify physical activity at the home environment in PD patients (Chastin et al., 2010). Nevertheless, no study has investigated the effect of any dance intervention on the activity level at home and in community. Furthermore, no DfPD study has objectively measured amount of activity (total limb acceleration) undertaken by people with PD during the class.
4. Quality of life (QoL) is increasingly recognised as a critical measure in health care in idiopathic PD and dance interventions have argued to be effective in enhancing QoL (Schrag, Jahanshahi, & Quinn, 2000). In addition, caring for a person with PD is associated with an increased risk of psychiatric morbidity and persistent distress. A recent systematic review points out notable lack of information regarding the quality of life of the patient and caregiver perception with related to dance interventions (Martinez-Martin et al., 2008).

***Section one***

# Research questions

1. Does the amount of activity (total limb acceleration) undertaken by people with PD during a DfP class related to their gait, balance and cognitive skills?

# Hypotheses

1. It is expected that the amount of activity (total limb acceleration) undertaken by people with PD during a DfP class is related to their gait, balance and cognitive skills.

***Section two***

# Research questions

1. Does dance for PD improve gait speed, stride length and step width during preferred pace walking in patients with PD?
2. Does dance for PD improve gait speed during dual tasking in patients with PD?
3. Does dance for PD improve executive function in patients with PD?
4. Does dance for PD lead to improved balance and coordination of the patient?
5. Does dance for PD lead to reduced caregiver burden?
6. Does dance for PD lead to improved QoL of the patient?
7. Does DfP programme leads to enhanced home activity level?
8. Is the effectiveness of DfP programme dependent on the amount of movement undertaken by the participant during the class?

# Hypotheses

1. It is expected that gait speed, stride length and step width of preferred pace walking will improve in response to dance for PD
2. It is expected that gait speed during dual tasking will improve in response to dance for PD
3. It is anticipated that executive function will improve in response to dance for PD
4. It is likely that dance for PD will contribute to improved balance and coordination
5. It is expected that dance for PD will lead to improved QoL of PD patient
6. It is anticipated that caregiver burden is reduced in response to dance for PD programme
7. It is expected that home activity level enhances in response to DfP.
8. The effectiveness of the DfP programme depends on the amount of movement undertaken by the participant during the class

For further details regarding the scales used in the study and the summarised study protocol, please refer appendix one.

The principle investigator is a physiotherapist and the supervisory team consists of a team of allied health professionals who have significant experience and expertise in working with individuals with PD.

# Has the scientific or academic merit of the research project been evaluated?

Review of the scientific or academic merit of the research project should be robust, formal and independent of the researcher team (e.g. a peer review of the protocol/proposal/research plan may have occurred at confirmation of candidature, or the researcher may have sought peer review from an independent scientist).

Peer review of the document was done by Dr. Toby Pavey, Research Ethics Advisor / Senior Lecturer, School of Exercise & Nutrition Sciences, Faculty of Health, QUT

# A1.5 Why should this be considered a negligible OR low risk application?

Refer to Chapter 2.1 of the National Statement when preparing your response and note that:

- ‘Negligible risk research’ describes research in which there is no foreseeable risk of harm or discomfort; and any foreseeable risk is no more than inconvenience (e.g. filling in a form, participating in a street survey, or giving up time to participate in research)**.**
- ‘Low risk research’ describes research in which the only foreseeable risk is one of discomfort (e.g. minor side-effects of medication, the discomforts related to measuring blood pressure, and anxiety induced by an interview).
- Research in which the risk for participants is more serious than discomfort (e.g. where a person’s reactions include pain or becoming distressed) the research cannot be considered low risk.

**A2 Potential Risks and Benefits** (refer to Chapter 2.1 of the National Statement when preparing your response)

A similar study with identical investigations, involving majority of the members of the supervisory panel, was approved by Human Research Ethics Committee of QUT, under low risk category in October 2013 (QUT Ethics Approval Number 1400000206).

The current application is considered low risk as it is proposed that no foreseeable risk of harm or discomfort beyond their normal daily experience of living with PD will occur. All clinical testing involving physical assessment activities is overseen by a team of allied health professionals who have significant experience and expertise in working with individuals with PD. Markers for signs of typical fatigue and balance issues caused by PD will be flagged and managed appropriately.

# A2.1 Describe ALL the identified potential risks and who may be affected by these risks e.g. researchers, participants,

**participant community and / or the wider community. Ensure all risks mentioned at A2.1 are addressed, and that the risks and their management are consistent throughout the application and are addressed where applicable in the Participant Information Sheet and Consent Form.**

When gauging the level of risk ensure you take into account:

- The kinds of harm, discomfort or inconvenience that may occur.
- The likelihood of these occurring.
- The severity of any harm that may occur.
- The choices, experience, perceptions, values and vulnerabilities of different populations of participants will also be relevant.

There is a possibility that participants may experience slight fatigue. There is also a slight risk that they may experience a loss of balance during some of the gait and balance-related tests. There is also a risk of falling, tripping and fatigue during the dance class. Application of ActivPALs using an adhesive tape may cause redness/itching in some participants. In addition if the participant has a hairy leg it may cause slight discomfort when removing the adhesive tape.

# A2.2 How are the risks to be minimised? And how will they be managed if they were to occur during the study or arise

**after the completion of the study?**

**NOTE:** The greater the risk to participants in any research for which ethical approval is given, the more certain it must be both that the risks will be managed as well as possible, and that the participants clearly understand the risks they are taking on. Ensure all risks mentioned at A2.1 are addressed here, that the risks and their management are consistent throughout the application and relevant information is included in the Participant Information Sheets and Consent Forms.

Risks to participants

- All tests will be administered across a set period of time providing adequate scheduled rest periods.
- During the assessments, participants are constantly monitored for any signs of tiredness or fatigue and are provided with frequent rest breaks.
- During the tests, participants are always closely monitored by at least one researcher (i.e. QUT or UQ) who is positioned in close proximity to prevent them from overbalancing.
- Participants are instructed that they can stop any test any time that they are uncomfortable with for any reason. They will never be forced to walk if they are anxious about the walkway.
- Persons who have the tendency to fall will be provided a harness support/walking sling during gait analysis.
- Questionnaires will be given for the participants to be taken home at the first visit, to be collected at a subsequent visit, to be completed in their own time. Adequate time (two weeks) will be given for the participants to complete and submit.
- ActivePALs will be applied with a non-allergy tape to minimize the possibility of causing any allergy. By any chance if participant experiences redness, pain or itching at the site where the tape contacts the skin, they will be advised to follow the instructions provided in the information sheet. In addition, if the participant is having a hairy leg, they will be advised to shave 10 X 10 cm on the centre of front thigh of one leg, prior to coming to the class. The area shaving needs to be done will be demonstrated in their first visit.
- Each dance class involves two dance teachers and a minimum two volunteers to make sure that each participant is closely monitored and to ensure their safety.
- During the designing of the DfP class, a lot of emphasis has been given for the safety of participants. 75 min dance classes start with 40 min seated warm up session followed by 15 min of standing dance techniques, and 20 min of moving across the floor. The maximum time allocated for seated dancing minimises the risk of

falling. 15 min standing dance is done while holding the back of a chair, so that it provides stability for the participant.

- Participants will be emphasised that they can continue as seated dancers if they wish to do so. One dancing teacher will be demonstrating seated dancing to facilitate seated participants.
- In order to further ensure their safety, the volunteers will be advised to pay special attention to participants who have increased a tendency to fall. If a participant looks suddenly tired, volunteers are advised to ask if he/she would like to sit down or take time out. Also, the volunteers are encouraged to get partnered to unstable participants during the partnered dance.

Risks to caregivers

- There will be no physical risks to caregivers. However when reflecting on the PD patient’s health condition, there may be some psychological impact on the caregiver, which may result in an emotional response. In this case, the researcher will show empathy and console the participant. There are professional services available in QUT, if they require help.

# A2.3 What are the potential benefits of the research and who would benefit from these?

- Benefits of research may include, e.g. gains in knowledge, insight and understanding, improved social welfare and individual wellbeing, and gains in skill or expertise for individual researchers, teams or institutions.
- Some research may offer direct benefits to the research participants, their families, or particular group/s with whom they identify. Where this is the case, participants may be ready to assume a higher risk than otherwise.

The QB DfP programme is designed to help people with a diagnosis of PD improve their quality of life through enhanced physical well-being, social interaction, creative expression, and targeted improvements in Parkinson’s symptoms including impaired balance, strength, and mobility, and cognitive impairment (Westheimer et al., 2015). It also focuses on the use of artistic interaction via designing the class programme around a QB performance season, the participation of company dancers in classes and attending live performances as a component of additional methods through which positive impacts can occur – beyond just participation in the physical dance classes.

Apart from a limited number of studies which deal with the effectiveness of different dance interventions on PD, to date, little work has been done to evaluate the effects of Dance for PD© programme, specifically on gait, dual tasking and cognition. Importantly, no prior study has conducted a detailed gait analysis using 3D motion analysis system, related to DfP program. Especially, it is rare to find dual tasking being addressed in past studies related to dance interventions. Therefore, the current study would fill the gap in knowledge regarding the effects of DfPD in the above mentioned areas. Furthermore, the current study would be important for DfP participants and caregivers to evaluate their improvement following the intervention. Moreover, this would fill the gap in knowledge related to the efficacy of DfP programme.

# A2.4 How do the benefits justify the risks?

- Research is ethically acceptable only when its potential benefits justify any risks involved in the research.

**A3 Other General Information**

The risks involved with this research are no greater than those encountered in everyday activities undertaken by people with PD. Even the 3D motion analysis of gait, which involves straight line walking with body markers, can be considered a normal everyday activity.

While these activities may cause slight fatigue for the participants, this will result in remarkable benefits in improving the condition. This dance for Parkinson’s programme offers the potential for enhanced physical well-being, social interaction, creative expression as well as improvements in strength, balance and walking (Westheimer et al., 2015).

**A3.1 Where will the data be collected?** (e.g. on site at QUT or other location)

**NOTE:** If you would like to conduct your study at the premises of an external organisation/association please ensure you provide a copy of your intended approach letter which requests their support/permission for this, or provide evidence of this if already gained.

**X**

**X**

# QUT Other – details:

Queensland Ballet Studios – Cnr Drake St. & Montague Rd. West End QLD 4101

**A3.2 Is the QUT Human Research Ethics Committee (UHREC) the primary or only ethics committee reviewing this proposal?**

If **NO**, provide details of any other institutional HREC involved and the role of each institution (including QUT) in the project. If the project involves more than one institution that also has a HREC, please provide details on the role of QUT UHREC; whether arrangements can be put in place for to minimise multiple review; arrangements for communication of the roles/responsibilities between the institutions HRECs, e.g. who will monitor etc.

YES

# A3.3 What are the estimated timeframes for the project? (mmm / yyyy)

**NOTE:** Data collection cannot commence until you have received formal written UHREC approval.

| **Start of project** | February 2016 | **Start of data collection** | January 2017 |
| --- | --- | --- | --- |
| **End of project** | February 2019 | **End of data collection** | August 2018 |

# A3.4 Describe the qualifications and relevant experience of the researcher team

**NOTE:** Include the training and experience student researchers have in the relevant research methodologies.

The QUT student researcher is a physiotherapist with past clinical experience with PD patients. The principal

**SECTION B: PARTICIPANT OVERVIEW** (refer to Chapter 2.2 of the National Statement when preparing your response)

supervisor, Prof. Graham Kerr, has extensive research experience and expertise in gait, and neural control of movement in PD. The Associate Supervisors Prof. Karen Sullivan is a Clinical Neuropsychologist, Prof. Sandy Brauer is an expertise in Physiotherapy, and A/Prof. Gene Moyle is an expert in Dance, and a Sport and Exercise Psychologist. In addition, Ms. Erica Rose Jeffrey who acts as an industry mentor, is the Programme Coordinator for Dance for Parkinson's Australia and she is a trained and registered teacher under in Dance for PD©.

# B1.1 Who will be approached to participate? Clearly outline each participant group.

Provide details of the potential participant pool. If you are accessing secondary data please provide full details, including whether permission has been sought. If you are accessing confidential health information e.g. Queensland Health data, the Public Health Act specifies the approvals required (see <http://www.health.qld.gov.au/ohmr/html/regu/aces_conf_hth_info.asp)> and QUT requires a Hospital Access Agreement. Contact the Division of Research & Commercialisation for assistance.

**Section one:** Participants will be recruited from those who are already involved in DfP programme at QB. They need to be individuals with a diagnosis of idiopathic PD (confirmed by a neurologist), aged 30-85 years, who are independently living, ambulatory and having the ability to walk independently for 10 feet without an assistive device. To be eligible to participate, people must be rated stage I-III on Hoehn and Yahr (1967) disability scale, indicating that they are not confined to bed or wheelchair. Moreover, they need to be within 24< in Mini Mental State Examination (MMSE) and 82< in Addenbrooke’s Cognitive Examination.

**Section two:** Participants will be individuals over the age of 30 to 85 years, independently living, ambulatory men or women who are diagnosed as having idiopathic PD. The diagnosis will be confirmed by their treating neurologist. In addition, they need to have the ability to walk independently for 10 feet without an assistive device, and stand for 30 min. To be eligible to participate, people must be rated stage I-III on Hoehn and Yahr (1967) disability scale, indicating that they are not confined to bed or wheelchair. Moreover, they need to be 24 < in Mini Mental State Examination (MMSE) and 82 < in Addenbrooke’s Cognitive Examination. These are widely accepted scores to exclude any cognitive impairment. In addition, they shouldn’t be participated in DfP programme for past six months. These participants will be randomly divided into experimental group and control group.

Consented caregivers of both, the intervention and control groups will also be recruited. Caregivers will be ‘‘any person who, without being a professional or belonging to a social support network, usually lives with the patient and, in some way, is directly implicated in the patient’s care or is directly affected by the patient’s health problem” (Martinez-Martin et al., 2008).

# B1.2 How many participants do you need for your study and approximately how many will you need to approach?

**Section one:** fifteen participants engaged in DfP class at QB will be recruited.

**Section two:** thirty participants with PD and thirty partners/caregivers will be allocated to the intervention group and a similar number of participants and care givers will be taken for the control group. (Sample size calculated using power analysis)

**B1.3 How will potential participants be identified and approached?**

**NOTE:** If you would like to recruit participants via an external organisation/association please ensure you provide a copy of your intended approach letter which requests their support/permission, or provide evidence of this if already gained.

**Section one:** Participants for the study section one will be initially contacted by the dance teachers who conduct the DfP class. Idiopathic PD patients who are currently participating in the DfP class, who consent to participate in the study will be recruited. Participants invited to meet with the researchers to formally review the Information Sheet and Informed Consent form. If consent is provided, they will be formally enrolled in the research project.

**Section two:** Idiopathic PD patients who have not involved in the DfP for past six months will be recruited through distributing recruitment flyers to the pre-existing DfP class at QB, flyers placed with allied health and medical practitioners, local advertisements, Parkinson’s Queensland Incorporated (PQI) at their website, newsletters and through contact with local PD support groups. Participants for this section two will be initially contacted by the allied health or medical practitioner or Nadeesha Kalyani (principal researcher).

# B1.4 How will the participants provide their consent to participate?

Outline the consent process you will use, what type of consent will be requested (i.e. specific, extended or unspecified – see NS2.2.14), what material will be provided to participants, how long participants will have to consider their decision to participate and what discussion will occur with participants.

**NOTE:**

- A person’s decision to participate in research must be voluntary and informed i.e. not forced, coerced or obtained by improper inducements AND based on sufficient information and adequate understanding of both the proposed research and the implications of participation in it (the purpose, methods, demands, risks and potential benefits of the research).
- The process of communicating information to participants and seeking their consent should not be merely a matter of satisfying a formal requirement. The aim is mutual understanding between researchers and participants. This aim requires an opportunity for participants to ask questions and to discuss the information and their decision with others if they wish.

Participants and the caregivers will be provided with Information Sheets (see attached) that outlines the purpose of the study, and the activities they will be required to complete. An Informed Consent Form will be reviewed in person with each of the participants.

# B1.5 Will the project involve participants who are unable to give voluntary or informed consent?

If **YES**, what special arrangements will be put in place to protect your participants’ interests/welfare? These questions refer to research involving:

- Children and young people whose particular level of maturity has implications for whether their consent is necessary and/or sufficient to authorise participation (see Chapter 4.2 of the National Statement).
- Persons with a cognitive impairment, and intellectual disability, or a mental illness (permanent or temporary) which impacts upon their ability to supply voluntary and informed consent (see Chapter 4.5 of the National Statement).
- Persons who are highly dependent on medical care, e.g. unconscious or unable to communicate their wishes (see Chapter 4.4 of the National Statement).
- Covert observation of behaviour, particularly if this relates to sensitive, contentious or illegal activity consent (see Chapter 2.3 and Chapter 4.6 of the National Statement).

**NOTE:** Where participants are unable to make their own decisions or have diminished capacity to do so, respect for them involves empowering them where possible and providing for their protection as necessary.

NO

**B1.6 Do you propose to screen or assess the suitability of the participants for the project?**

If **YES**, clearly state and explain the criteria (inclusion and exclusion, as applicable) for selecting potential participants.

YES.

# Section one:

Inclusion criteria: Individuals with a diagnosis of idiopathic PD (confirmed by a neurologist) who are independently living, ambulatory and able to walk independently for 10 feet without an assistive device. To be eligible to participate, people must be rated stage I-III on the Hoehn and Yahr (1967) disability scale, indicating that they are not confined to bed or wheelchair. Moreover, they need to be higher than 24 in the Mini Mental State Examination (MMSE) and higher than 82 in Addenbrooke’s Cognitive Examination.

Exclusion criteria: Those who have a Mini Mental State Examination score less than 24 and an Addenbrooke’s Cognitive Examination score less than 82, with confounding medical (e.g. severe blood pressure abnormalities), neurological (e.g. stroke), musculoskeletal (e.g. recent musculoskeletal injuries, fractures or surgeries), cardiovascular (e.g. recent cardiac surgeries) or respiratory abnormalities (e.g. Chronic Obstructive Pulmonary Disease, asthma) will be excluded.

# Section two:

Inclusion criteria: Individuals with a diagnosis of idiopathic PD (confirmed by a neurologist), who are able and willing to commit to attending DfP classes consecutively twice a week for twelve weeks, will be included in this study. Participants need to be independently living, ambulatory men or women with an ability to walk independently for 10 feet without an assistive device. To be eligible to participate, people must be rated stage I-IV on the Hoehn and Yahr (1967) disability scale, indicating that they are not confined to bed or wheelchair. Moreover, they need to be higher than 24 in the Mini Mental State Examination (MMSE) and higher than 82 in Addenbrooke’s Cognitive Examination.

Exclusion criteria: Participants who are currently engaged or have engaged in DfP classes during the past 6 months will be excluded. In addition, those with a Mini Mental State Examination score of less than 24 and Addenbrooke’s Cognitive Examination of less than 82 with confounding medical (e.g. severe blood pressure abnormalities), neurological (e.g. stroke), musculoskeletal (e.g. recent musculoskeletal injuries, fractures or surgeries), cardiovascular (e.g. recent cardiac surgeries) or respiratory abnormalities (e.g. Chronic Obstructive Pulmonary Disease, asthma) will be excluded.

The potential participant who does not meet the screening criteria and who is not eligible for the study, will still be given a written report which contains basic information on their gait, balance and cognitive skills.

Consenting caregivers of both intervention and control groups will also be recruited.

# B1.7 Will participants be offered reimbursements, payments or incentives?

If **YES**, also provide the specific details (type and value), how and when it will be provided and whether its offer could compromise the voluntary nature of the consent obtained from participants. See Guidance on prize draws.

**NOTE:** Details of these should be provided on the Participant Information Sheet.

- It is generally appropriate to reimburse the costs to participants of taking part in research, including costs such as travel, accommodation and parking. Sometimes participants may also be paid for time involved. However, payment that is disproportionate to the time involved, or any other inducement that is likely to encourage participants to take risks, is ethically unacceptable (NS2.2.10)
- Decisions about payment or reimbursement in kind, whether to participants or their community, should take into account the customs and practices of the community in which the research is to be conducted (NS2.2.11)

Participants of the study-section two will be offered free parking at QUT during the assessments.

# B1.8 Do you, or others involved in facilitating or implementing the research, have a pre-existing relationship with the

**proposed participants? Could this result in the proposed participants feeling obliged or coerced into participation?** Refer to Chapter 4.3 of the National Statement and the QUT Research Data Collection in Classrooms or Lecture Theatres guidance when considering/preparing your response.

If **YES**, describe this relationship and how you will address the special ethical issues this raises (e.g. potential coercion in recruitment). Also outline what special arrangements will be put in place to protect the interests / welfare of potential participants. **NOTE:**

- Pre-existing relationships may compromise the voluntary nature of participants’ decisions, as they typically involve unequal

status, where one party has or has had a position of influence or authority over the other.

- Examples may include relationships between employers or supervisors and their employees; teachers and their students; carers and people with chronic conditions or disabilities or people in residential care or supported accommodation; etc (see Chapter 4.3 of the National Statement for more examples).
- While this influence does not necessarily invalidate the decision, it does mean that particular attention should be given to the process through which consent is negotiated.

Participants involved in the study-part 1 already have a pre-existing relationship with the dancing instructor, principle investigator and QB, since they are currently attending the DfP program.

It is anticipated that participants involved in the study- part 2, will not have any pre-existing relationship with QUT, UQ, QB or any of the investigators of the study.

It will be highlighted to all potential participants that their participation in the current study is completely voluntary, that there is no expectation of involvement, and that should they decide to participate they can still withdraw from the project at any time. They will be encouraged to discuss their participation with their caregivers and/or support network prior to making a decision. Furthermore, it will also be highlighted to potential participants that choosing not to participate will have no bearing or impact upon the relationship with QUT, UQ, or QB and their involvement DfP classes prior to the completion of the program.

# B1.9 Will you conduct a debriefing session at the end of the research or at the end of each participant’s involvement?

If **YES**, please provide the details of this session. **NOTE:** Such a session is required for research involving deception (see Chapter 2.3 of the National Statement), and may be appropriate if the research is likely to cause discomfort to participants.

NO.

# B1.10 Consider providing feedback to participants as this is encouraged by the National Statement.

**Will feedback and/or the research results be reported to participants?**

- If **YES**, explain how this will be done and in what form this reporting will occur.
- If **NO**, explain why the participants are not to be provided with such a report.

**SECTION C: DATA MANAGEMENT**

**C1 Future Use of Data**

YES. Participants will be provided with a general summary report based on aggregate results at the conclusion of the project. They will be provided with more in-depth knowledge about their condition. This will include disease severity, falls risk, gait, balance and cognitive skills.

If the participant requests, a report of the assessment can be sent to the treating doctor. The report will contain disease severity, falls risk, gait, balance and the cognitive skills of the participant.

**C1.1 Is it likely / possible that any of the data collected will be used by you, or others, for any research other than that outlined in this application?** See Chapter 2.2 and Chapter 3.2 when preparing your response.

**If YES, describe below and ensure this is outlined in all your participant information sheets and consent forms.**

- Participants should be fully informed of the possibility of any future use of data collected and their ‘extended’ or ‘unspecified’ consent gained. Failure to do this may restrict the future use of the data.
- Any restrictions on the use of participants’ data should be recorded and the record kept with the collected data so that it is always accessible to researchers who want to access those data for research.
- Please note that data sharing is increasingly being encouraged to gain maximum benefit from research, so a **YES** response is encouraged in most cases. If **YES**, describe below and ensure this is outlined in all your Participant Information Sheets and Consent Forms.

**C2 Procedures & Protection**

YES. The data collected in the study may be utilised for future research to further investigate the impacts of participation in DfP programs. Furthermore, a summary of the findings is likely to be utilised as part of future funding applications for grants to further expand and progress this initial research.

# C2.1 What data collection procedures will be utilised?

Place an ‘**X**’ in the relevant boxes below **AND** provide a copy (draft or finalised, labelled as such) of the relevant instrument, protocol or other written form used to guide (e.g. interview questions/guide) or collect data (e.g. survey) or include an explanation of the method by which the data will be collected. Clinical experimental measures / tools or creative works are considered “Other Instrument”.

| **X** | **Questionnaires/Surveys Archival records**  **Interviews Focus groups**  **Other instrument – provide details:** (If there is insufficient space below, provide details in an additional separate document) |
| --- | --- |
| **X** |  |
| **X** |  |
| Interviews, Questionnaires, Audio/Videotaping, Participant Diaries, Clinical Instruments | |

**C2.2 Have the data collection procedures been previously approved by QUT or are they an academic standard instrument?** If **YES**, provide brief details on prior approval or where instruments have been used previously, e.g. under a similar research context

YES.

Previous pilot study on Dance for Parkinson’s (QUT Ethics Approval Number 1400000206).

# C2.3 In what form will the human data be collected, stored and used/reported?

In each row, indicate which form of data (eg. interviews, questionnaires etc) applies for your study.

**At least one column must be completed in each row but if different data are in different forms, you will need to complete more**

**than one box in each column or row.**

|  | **Individually Identifiable**  i.e. Data from which the identity of a specific individual can be reasonably ascertained eg. name,  image, date of birth, and/or address. | **Re-Identifiable or Potentially Re-Identifiable**  i.e. Data from which identifiers have been removed and replaced by codes, but it remains possible to re-identify individuals, eg. by using  the code or linking different data sets. | **Non-Identifiable**  i.e. Data that have never been labelled with individual identifiers OR from which identifiers have been permanently removed such that no specific individual can be identified by the researchers. |
| --- | --- | --- | --- |
| **Collected** | Semi-structured interviews, Questionnaires, Clinical Measurements, Audio/Video, Diaries |  |  |
| **Stored** | Audio recorded Semi-structured interviews /Videos of the dance  classes | Questionnaires, Clinical Measurements, Diaries |  |
| **Used/Reported** | Audio recorded Semi-structured interviews /Videos of the dance  classes |  | Questionnaires, Clinical Measurements, Diaries |

# C2.4 How is this project funded?

Outline what rights the funder of the study will have to data obtained from the study, and in what format e.g. aggregate reports only, access to raw data or other. **NOTE:** Any access by the funder should be made clear to participants.

NO.

# C2.5 How will confidentiality of the study records be protected during the study and in the publication of results?

**NOTE:** If you intend to identify participants or organisations, this needs to be made clear on the Participant Information Sheet.

All participants will be made aware of the nature of the research and its proposed outcomes. Informed consent will be sought from each participant to provide them the opportunity to participate and identify themselves within the framework of the research. Unless otherwise indicated, all participants will remain anonymous. Participants will be provided with an identity code which will be used for collation of data and subsequent data analysis. No individuals will be identified in scientific publications resulting from the project. In order to further ensure the confidentiality, good storage practices will be incorporated including locked filing cabinets and password protected files etc.

# C2.6 Is this a collaborative project?

If **YES**, also provide brief detail on data-sharing arrangements e.g. open – all parties have access to each other’s data; partial – data held by collaborator completing particular component.

NO.

**C2.7 Who will own the resulting research data and the created intellectual property?**

Place an ‘**X**’ in the relevant box/es below – at least one box must be checked. If relevant you can check more than one box, ie QUT

and an external organisation. Please refer to the D/3.1 Intellectual property (IP) policy for further information.

|  |
| --- |
| **X** |
|  |

**QUT** – QUT is the owner of IP created by staff members in the course of their employment.

**STUDENT/S** –The IP generated is personally owned by the student if not assigned to QUT or other organisation.

**BOTH QUT & STUDENT/S** – If the IP for a student project has been assigned to QUT, ownership of data and IP is shared (see Student IP protocol).

**EXTERNAL ORGANISATION** – Give details:

**NOTE:** QUT requires an IP agreement to be in place if IP ownership is to deviate from that described in D/3.1 Intellectual property (IP) policy. Please contact the relevant section of the Division of Research & Commercialisation if you require any further assistance.

**C3 Storage & Security**

Ensure you have completed your QUT Data Management Plan **BEFORE** completing this section.

- Data should be stored in a locked filing cabinet at QUT and/or electronically on a QUT mainframe drive.
- Data must not be stored solely at home.

# C3.1 C3.2

**YES Confirm that your research data and other records will be stored for the required period.**

Refer to the Management research data.

**X**

**HARD/PAPER COPIES...** (e.g. **signed consent forms**, are required to be kept for **15 years** as per the Qld State Archives Schedule) Qld State Archives: <http://www.archives.qld.gov.au/Recordkeeping/Governance/Pages/Default.aspx>

University Sector: <http://www.archives.qld.gov.au/Recordkeeping/GRKDownloads/Documents/Universities.pdf>

| **C3.2.1 What is the location/s of storage?**  (i.e. QUT room/building location and/or offsite storage location) | A central file server with log-on access will be provided for researcher access only. |
| --- | --- |
| **C3.2.2 How will access to the stored data be controlled?** | Lockable physical storage equipment will be utilised for hardcopy data. Authorised access will be managed via the use of a signed confidentially/non-disclosure  agreement. |

# C3.2.3 Who will have access to the stored data? C3.3 ELECTRONIC DATA...

| Only the researchers. |
| --- |
|  |
| Data will be stored on password-protected computers connected to servers at each of the researcher’s institutions. Secure Log-on access will be provided. The data will have a guard against unauthorised access by using physical (locks) or digital (encryption/password)  controls. |
| Only researchers will have access to the data. |
| Only the researchers. |

**C3.3.1 What is the location/s of storage and back-up?**

(i.e. a secure computer/server and/or offsite storage location)

**C3.3.2 How will access to the stored data be controlled? C3.3.3 Who will have access to the stored data?**
