# Supplementary material for: Effects of dance on gait and dual-task gait in Parkinson’s disease
Source: PLoS One. 2023 Jan 24;18(1):e0280635. doi: 10.1371/journal.pone.0280635 (PMC9873168; doi:10.1371/journal.pone.0280635)
Supplement: S2 File — (DOCX) [file pone.0280635.s004.docx]

***Annex 1:*** *Baseline comparison of dance group and control group: spatiotemporal parameters of gait during even surface walking*

| *Spatiotemporal variables* | *Normal walking*  *Mean ± S.E* | | | *Serial subtraction*  *Mean ± S.E* | | | *Verbal Fluency*  *Mean ± S.E* | | |
| --- | --- | --- | --- | --- | --- | --- | --- | --- | --- |
|  | ***Dance***  ***(n=17)*** | ***Control***  ***(n=16)*** | ***p*-value** | ***Dance***  ***(n=17)*** | ***Control***  ***(n=16)*** | ***p*-value** | ***Dance***  ***(n=17)*** | ***Control***  ***(n=16)*** | ***p*-value** |
| Gait velocity (ms^-1^) | 1.04  (0.04) | 0.98  (0.04) | 0.38 | 0.83  (0.05) | 0.83  (0.04) | 0.86 | 0.85  (0.04) | 0.82  (0.04) | 0.73 |
| Cadence (steps/sec.) | 1.80  (0.04) | 1.80  (0.04) | 0.88 | 1.63  (0.05) | 1.66  (0.05) | 0.54 | 1.66  (0.06) | 1.66  (0.06) | 0.76 |
| Step length (metres) | 0.57  (0.02) | 0.54  (0.01) | 0.27 | 0.50  (0.02) | 0.50  (0.02) | 1.00 | 0.51  (0.02) | 0.49  (0.02) | 0.61 |
| Stride length (metres) | 1.14  (0.03) | 1.08  (0.03) | 0.27 | 1.01  (0.43) | 0.99  (0.04) | 0.99 | 1.02  (0.04) | 0.98  (0.04) | 0.55 |
| Stance phase (%) | 63.71  (0.54) | 64.40  (0.50) | 0.26 | 65.22  (0.58) | 66.05  (0.53) | 0.76 | 62.47  (0.86) | 63.64  (0.79) | 0.33 |
| Swing phase (%) | 36.29  (0.54) | 33.59  (0.49) | 0.26 | 34.78  (0.58) | 33.95  (0.53) | 0.76 | 37.53  (0.86) | 36.35  (0.79) | 0.33 |
| Double support (%) | 27.42  (1.09) | 28.81  (1.00) | 0.26 | 30.44  (1.16) | 32.09  (1.06) | 0.76 | 24.94  (1.73) | 27.29  (1.58) | 0.33 |
| Single support (%) | 72.58  (1.09) | 71.19  (1.00) | 0.26 | 69.56  (1.16) | 67.91  (1.06) | 0.76 | 75.15  (1.71) | 72.67  (1.59) | 0.33 |
| Stride time variability | 36.57  (27.78) | 26.78  (10.71) | 0.97 | 73.50  (45.07) | 37.90  (17.06) | 0.05 | 56.61  (43.23) | 49.36  (27.07) | 0.63 |

SE = standard error, Linear Mix Model applied for the baseline measures of dance and control groups. Covariates: gender and disease severity. Stride time variability is the standard deviation of the stride period. A *p*-value < 0.05 is considered as significant.

***Annex II:*** *Baseline comparison of dance group and control group: spatiotemporal parameters of gait during uneven surface walking*

| *Spatiotemporal variables* | *Normal walking*  *Mean ± S.E* | | | *Serial subtraction*  *Mean ± S.E* | | | *Verbal Fluency*  *Mean ± S.E* | | |
| --- | --- | --- | --- | --- | --- | --- | --- | --- | --- |
|  | ***Dance***  ***(n=17)*** | ***Control***  ***(n=16)*** | ***p*-value** | ***Dance***  ***(n=17)*** | ***Control***  ***(n=16)*** | ***p*-value** | ***Dance***  ***(n=17)*** | ***Control***  ***(n=16)*** | ***p*-value** |
| Gait velocity (ms^-1^) | 0.91  (0.06) | 0.86  (0.06) | 0.52 | 0.70  (0.05) | 0.75  (0.05) | 0.73 | 0.75  (0.05) | 0.70  (0.05) | 0.58 |
| Cadence (steps/sec.) | 1.66  (0.06) | 1.66  (0.06) | 0.90 | 1.45  (0.05) | 1.54  (0.05) | 0.31 | 1.47  (0.06) | 1.51  (0.06) | 0.79 |
| Step length (metres) | 0.51  (0.02) | 0.49  (0.02) | 0.37 | 0.54  (0.02) | 0.51  (0.02) | 0.94 | 0.48  (0.02) | 0.48  (0.02) | 0.35 |
| Stride length (metres) | 1.02  (0.04) | 0.98  (0.04) | 0.35 | 1.08  (0.05) | 1.02  (0.05) | 0.86 | 0.95  (0.05) | 0.95  (0.05) | 0.39 |
| Stance phase (%) | 62.46  (0.86) | 63.65  (0.79) | 0.15 | 65.31  (1.00) | 64.52  (0.91) | 0.69 | 64.50  (0.82) | 65.30  (0.75) | 0.46 |
| Swing phase (%) | 37.53  (0.86) | 36.35  (0.79) | 0.15 | 34.69  (1.00) | 35.48  (0.91) | 0.69 | 35.50  (0.82) | 34.70  (0.75) | 0.46 |
| Double support (%) | 24.94  (1.73) | 27.29  (1.58) | 0.15 | 30.62  (2.00) | 29.04  (1.83) | 0.69 | 29.00  (1.64) | 30.60  (1.50) | 0.46 |
| Single support (%) | 75.13  (1.71) | 72.67  (1.57) | 0.19 | 66.38  (2.00) | 70.96  (1.83) | 0.53 | 70.99  (1.64) | 69.39  (1.68) | 0.65 |
| Stride time variability | 78.08  (74.96) | 53.12  (37.00) | 0.60 | 138.33  (112.27) | 74.42  (40.71) | 0.40 | 116.12  (109.33) | 79.77  (81.99) | 0.19 |

SE = standard error, Linear Mix Model applied for the baseline measures of dance and control groups. Covariates: gender and disease severity. Stride time variability is the standard deviation of the stride period. A *p*-value < 0.05 is considered as significant.

***Annex III:*** *Post-pre comparison: Spatiotemporal parameters of gait during regular walking on even surface*

| *Walking conditions* | *Pre-test^1^*  *Mean ± S.E* | | *Post-test*  *Mean ± S.E* | | *Change scores Mean^2^*  *[95% CI]* | | *p-value^3^* | *Effect size^4^* |
| --- | --- | --- | --- | --- | --- | --- | --- | --- |
|  | *Dance*  *(n=17)* | *Control*  *(n=16)* | *Dance*  *(n=17)* | *Control*  *(n=16)* | *Dance*  *(n=17)* | *Control*  *(n=16)* |  |  |
| Gait velocity (ms^-1^) | 1.04  (0.04) | 0.98  (0.04) | 1.19  (0.04) | 0.99  (0.04) | 0.150  [0.075, 0.226] | 0.013  [-0.056, 0.082] | **p = 0.017** | 0.976 |
| Cadence (steps/sec.) | 1.80  (0.04) | 1.80  (0.04) | 1.95  (0.04) | 1.82  (0.04) | 0.120  [0.053,0.188] | 0.015  [-0.046,0.077] | **p = 0.039** | 0.084 |
| Step length (metres) | 0.57  (0.02) | 0.54  (0.01) | 0.61  (0.02) | 0.55  (0.01) | 0.044  [0.018,0.070] | 0.004  [-0.020,0.027] | **p = 0.040** | 0.809 |
| Stride length (metres) | 1.14  (0.03) | 1.08  (0.03) | 1.22  (0.03) | 1.09  (0.03) | 0.088  [0.036,0.140] | 0.01  [-0.039,0.055] | **p = 0.041** | 0.843 |
| Stance phase (%) | 63.71  (0.54) | 64.40  (0.50) | 62.74  (0.54) | 64.35  (0.50) | -0.745  [-1.574,0.084] | -0.144  [-0.905,0.618] | p = 0.324 | 0.573 |
| Swing phase (%) | 36.29  (0.54) | 33.59  (0.49) | 37.25  (0.54) | 35.65  (0.49) | 0.745  [-0.084,1.574] | 0.144  [-0.618,0.905] | p = 0.324 | 0.573 |
| Double support (%) | 27.42  (1.09) | 28.81  (1.00) | 25.50  (1.09) | 28.71  (1.00) | -1.490  [-3.147,0.168] | -0.288  [-1.811,1.235] | p = 0.324 | 0.045 |
| Single support (%) | 72.58  (1.09) | 71.19  (1.00) | 74.50  (1.09) | 71.29  (1.00) | 1.490  [-0.168,3.147] | 0.288  [-1.235,1.811] | p = 0.324 | 0.045 |
| Stride time variability | 36.57  (27.78) | 26.78  (10.71) | 23.33  (19.50) | 26.84  (15.18) | -13.24  [-24.08, -2.40] | -0.34  [-7.11, 6.43] | p = 0.249 | 0.743 |

SE = standard error, ^1^ Dance and control group were not significantly different for any measure at baseline, ^2^ Linear Mix Model applied for change scores. Covariates: gender and disease severity. Change score represents mean [95% CI], ^3^ Bold values are significant (*p*-value < 0.05), ^4^Effect sizes (d) calculated using Campbell Collaboration online calculator

***Annex IV:*** *Post-pre comparison: Spatiotemporal parameters of gait during dual tasking (performance of a verbal fluency task) on even surface*

| *Walking conditions* | *Pre-test^1^*  *Mean ± S.E* | | *Post-test*  *Mean ± S.E* | | *Change scores Mean^2^*  *[95% CI]* | | *p-value^3^* | *Effect size^4^* |
| --- | --- | --- | --- | --- | --- | --- | --- | --- |
|  | *Dance*  *(n=17)* | *Control*  *(n=16)* | *Dance*  *(n=17)* | *Control*  *(n=16)* | *Dance*  *(n=17)* | *Control*  *(n=16)* |  |  |
| Gait velocity (ms^-1^) | 0.85  (0.04) | 0.82  (0.04) | 0.99  (0.04) | 0.83  (0.04) | 0.140  [0.061, 0.22] | 0.015  [-0.056, 0.086] | **p = 0.035** | 0.866 |
| Cadence (steps/sec.) | 1.66  (0.06) | 1.66  (0.06) | 1.77  (0.06) | 1.67  (0.06) | 0.136  [0.067, 0.207] | 0.024  [-0.041, 0.089] | **p = 0.034** | 0.672 |
| Step length (metres) | 0.51  (0.02) | 0.49  (0.02) | 0.55  (0.02) | 0.49  (0.02) | 0.041  [0.015, 0.068] | 0.0001  [-0.024,0.025] | **p = 0.039** | 0.828 |
| Stride length (metres) | 1.02  (0.04) | 0.98  (0.04) | 1.11  (0.04) | 0.99  (0.04) | 0.083  [0.03, 0.137] | 0.006  [-0.043, 0.055] | **p = 0.053** | 0.779 |
| Stance phase (%) | 62.47  (0.86) | 63.64  (0.79) | 62..55  (0.86) | 63.11  (0.80) | 0.093  [-0.813, 0.999] | 0.141  [0.707, 0.989] | p = 0.942 | 0.028 |
| Swing phase (%) | 37.53  (0.86) | 36.35  (0.79) | 37.44  (0.86) | 36.89  (0.80) | -0.093  [-0.999, 0.813] | -0.141  [-0.989, 0.707] | p = 0.942 | 0.028 |
| Double support (%) | 24.94  (1.73) | 27.29  (1.58) | 25.11  (1.73) | 26.22  (1.60) | 0.186  [-1.626, 1.997] | 0.282  [-1.415, 1.979] | p = 0.942 | 0.028 |
| Single support (%) | 75.15  (1.71) | 72.67  (1.59) | 74.59  (1.71) | 73.26  (1.58) | -0.186  [-1.997, 1.626] | -0.282  [-1.979, 1.415] | p = 0.942 | 0.028 |
| Stride time variability | 56.61  (43.23) | 49.36  (27.07) | 35.42  (25.05) | 41.23  (18.33) | -21.19  [-46.58, 4.21] | -8.13  [-24.22, 7.96] | p = 0.272 | 0.318 |

SE = standard error, ^1^ Dance and control group were not significantly different for any measure at baseline, ^2^ Linear Mix Model applied for change scores. Covariates: gender and disease severity. Change score represents mean [95% CI], ^3^ Bold values are significant (*p*-value < 0.05), ^4^Effect sizes (d) calculated using Campbell Collaboration online calculator

***Annex V:*** *Post-pre comparison: Spatiotemporal parameters of gait during dual tasking (performance of a serial subtraction task) on even surface*

| *Walking conditions* | *Pre-test^1^*  *Mean ± S.E* | | *Post-test*  *Mean ± S.E* | | *Change scores Mean^2^*  *[95% CI]* | | *p-value^3^* | *Effect size^4^* |
| --- | --- | --- | --- | --- | --- | --- | --- | --- |
|  | *Dance*  *(n=17)* | *Control*  *(n=16)* | *Dance*  *(n=17)* | *Control*  *(n=16)* | *Dance*  *(n=17)* | *Control*  *(n=16)* |  |  |
| Gait velocity (ms^-1^) | 0.83  (0.05) | 0.83  (0.04) | 0.97  (0.05) | 0.99  (0.04) | 0.146  [0.069, 0.224] | -0.003  [-0.075, 0.068] | **p = 0.012** | 1.033 |
| Cadence (steps/sec.) | 1.63  (0.05) | 1.66  (0.05) | 1.78  (0.05) | 1.67  (0.05) | 0.123  [0.053, 0.193] | 0.0001  [-0.064, 0.065] | **p = 0.021** | 0.905 |
| Step length (metres) | 0.50  (0.02) | 0.50  (0.02) | 0.55  (0.02) | 0.50  (0.02) | 0.048  [0.022, 0.075] | 0.001  [-0.024,0.025] | **p = 0.018** | 0.951 |
| Stride length (metres) | 1.01  (0.43) | 0.99  (0.04) | 1.10  (0.43) | 1.00  (0.04) | 0.097  [0.044, 0.151] | -0.001  [-0.050, 0.048] | **p = 0.015** | 0.992 |
| Stance phase (%) | 65.22  (0.58) | 66.05  (0.53) | 65.13  (0.58) | 66.23  (0.53) | 0.498  [-1.399, 0.403] | 0.176  [-0.669, 1.021] | p = 0.311 | 0.392 |
| Swing phase (%) | 34.78  (0.58) | 33.95  (0.53) | 34.87  (0.58) | 33.77  (0.53) | -0.498  [-0.403, 1.399] | -0.176  [-1.021, 0.669] | p = 0.311 | 0.392 |
| Double support (%) | 30.44  (1.16) | 32.09  (1.06) | 30.26  (1.16) | 32.46  (1.06) | 0.996  [-2.798, 0.807] | 0.352  [-1.338, 2.041] | p = 0.311 | 0.392 |
| Single support (%)  Stride time variability | 69.56  (1.16)  73.50  (45.07) | 67.91  (1.06)  37.90  (17.06) | 69.74  (1.16)  55.00  (42.23) | 67.53  (1.06)  36.86  (17.23) | -0.996  [-0.807, 2.798]  -18.50  [-42.89, 5.89] | -0.352  [-2.041, 1.338]  -1.04  [-12.75, 10.67] | p = 0.311  p = 0.318 | 0.392  0.470 |

SE = standard error, ^1^ Dance and control group were not significantly different for any measure at baseline, ^2^ Linear Mix Model applied for change scores. Covariates: gender and disease severity. Change score represents mean [95% CI], ^3^ Bold values are significant (*p*-value < 0.05), ^4^Effect sizes (d) calculated using Campbell Collaboration online calculator

***Annex VI:*** *Post-pre comparison: Spatiotemporal parameters of gait during regular walking on uneven surface*

| *Walking conditions* | *Pre-test*  *Mean ± S.E* | | *Post-test*  *Mean ± S.E* | | *Change scores Mean^2^*  *[95% CI]* | | *p-value^3^* | *Effect size^4^* |
| --- | --- | --- | --- | --- | --- | --- | --- | --- |
|  | *Dance*  *(n=17)* | *Control*  *(n=16)* | *Dance*  *(n=17)* | *Control*  *(n=16)* | *Dance*  *(n=17)* | *Control*  *(n=16)* |  |  |
| Gait velocity (ms^-1^) | 0.91  (0.06) | 0.86  (0.06) | 1.05  (0.06) | 0.89  (0.06) | 0.130  [0.046,0.214] | 0.040  [-0.039,0.12] | p = 0.162 | 0.570 |
| Cadence (steps/sec.) | 1.66  (0.06) | 1.66  (0.06) | 1.76  (0.06) | 1.67  (0.06) | 0.096  [0.011,0.181] | 0.010  [-0.071,0.092] | p = 0.186 | 0.531 |
| Step length (metres) | 0.51  (0.02) | 0.49  (0.02) | 0.55  (0.02) | 0.49  (0.02) | 0.045  [0.012,0.078] | 0.020  [-0.012,0.051] | p = 0.307 | 0.396 |
| Stride length (metres) | 1.02  (0.04) | 0.98  (0.04) | 1.11  (0.04) | 0.99  (0.04) | 0.091  [0.028,0.155] | 0.050  [-0.01,0.111] | p = 0.392 | 0.347 |
| Stance phase (%) | 62.46  (0.86) | 63.65  (0.79) | 62.55  (0.86) | 63.11  (0.80) | 0.843  [-0.372,2.059] | -1.002  [-2.189,0.185] | **p = 0.049** | 0.067 |
| Swing phase (%) | 37.53  (0.86) | 36.35  (0.79) | 37.44  (0.86) | 36.89  (0.80) | -0.843  [-2.059,0.372] | 1.076  [-0.139,2.292] | **p = 0.049** | 0.067 |
| Double support (%) | 24.94  (1.73) | 27.29  (1.58) | 25.11  (1.73) | 26.22  (1.60) | 1.687  [-0.744,4.118] | -2.003  [-4.377,0.37] | **p = 0.049** | 0.417 |
| Single support (%) | 75.13  (1.71) | 72.67  (1.57) | 74.59  (1.71) | 73.26  (1.58) | -1.687  [-4.118,0.744] | 2.003  [-0.37,4.377] | **p = 0.049** | 0.417 |
| Stride time variability | 78.08  (74.96) | 53.12  (37.00) | 64.61  (87.13) | 45.30  (25.57) | -13.47  [-57.95, 31.00] | -7.82  [-25.37, 9373] | p = 0.434 | 0.085 |

SE = standard error, ^1^ Dance and control group were not significantly different for any measure at baseline, ^2^ Linear Mix Model applied for change scores. Covariates: gender and disease severity. Change score represents mean [95% CI], ^3^ Bold values are significant (*p*-value < 0.05), ^4^Effect sizes (d) calculated using Campbell Collaboration online calculator

| *Walking conditions* | *Pre-test^1^*  *Mean ± S.E* | | *Post-test*  *Mean ± S.E* | | *Change scores Mean^2^*  *[95% CI]* | | *p-value^3^* | *Effect size^4^* |
| --- | --- | --- | --- | --- | --- | --- | --- | --- |
|  | *Dance*  *(n=17)* | *Control*  *(n=16)* | *Dance*  *(n=17)* | *Control*  *(n=16)* | *Dance*  *(n=17)* | *Control*  *(n=16)* |  |  |
| Gait velocity (ms^-1^) | 0.75  (0.05) | 0.70  (0.05) | 0.90  (0.05) | 0.74  (0.05) | 0.138  [0.054, 0.222] | 0.046  [-0.034, 0.126] | p = 0.152 | 0.583 |
| Cadence (steps/sec.) | 1.47  (0.06) | 1.51  (0.06) | 1.64  (0.06) | 1.54  (0.06) | 0.152  [0.067, 0.237] | 0.042  [-0.040, 0.124] | p = 0.092 | 0.672 |
| Step length (metres) | 0.48  (0.02) | 0.48  (0.02) | 0.55  (0.02) | 0.49  (0.02) | 0.046  [0.013, 0.079] | 0.011  [-0.021, 0.043] | p = 0.174 | 0.555 |
| Stride length (metres) | 0.95  (0.05) | 0.95  (0.05) | 1.08  (0.05) | 0.99  (0.05) | 0.092  [0.029, 0.155] | 0.027  [-0.034, 0.088] | p = 0.182 | 0.540 |
| Stance phase (%) | 64.50  (0.82) | 65.30  (0.75) | 62.67  (0.82) | 64.72  (0.76) | -1.076  [-2.292, 0.139] | -1.194  [-2.397, 0.009] | p = 0.899 | 0.050 |
| Swing phase (%) | 35.50  (0.82) | 34.70  (0.75) | 37.33  (0.82) | 35.28  (0.76) | 1.076  [-0.139, 2.292] | 1.194  [-0.009, 2.397] | p = 0.899 | 0.050 |
| Double support (%) | 29.00  (1.64) | 30.60  (1.50) | 25.34  (1.64) | 29.45  (1.53) | -2.152  [-4.584, 0.279] | -2.388  [-4.795, 0.019] | p = 0.899 | 0.049 |
| Single support (%) | 70.99  (1.64) | 69.39  (1.68) | 74.66  (1.64) | 70.55  (1.53) | 2.152  [-0.279, -0.019] | 2.388  [-0.019, 4.795] | p = 0.899 | 0.049 |
| Stride time variability | 116.12  (109.33) | 79.77  (81.99) | 62.48  (32.58) | 90.76  (100.63) | -53.64  [-105.05, -2.23] | 10.99  [-40.96, 62.95] | p = 0.286 | 0.666 |

***Annex VII:*** *Post-pre comparison: Spatiotemporal parameters of gait during dual tasking (performance of a verbal fluency task) on uneven surface*

SE = standard error, ^1^ Dance and control group were not significantly different for any measure at baseline, ^2^ Linear Mix Model applied for change scores. Covariates: gender and disease severity. Change score represents mean [95% CI], ^3^ Bold values are significant (*p*-value < 0.05), ^4^Effect sizes (d) calculated using Campbell Collaboration online calculator

| *Walking conditions* | *Pre-test^1^*  *Mean ± S.E* | | *Post-test*  *Mean ± S.E* | | *Change scores Mean^2^*  *[95% CI]* | | *p-value^3^* | *Effect size^4^* |
| --- | --- | --- | --- | --- | --- | --- | --- | --- |
|  | *Dance*  *(n=17)* | *Control*  *(n=16)* | *Dance*  *(n=17)* | *Control*  *(n=16)* | *Dance*  *(n=17)* | *Control*  *(n=16)* |  |  |
| Gait velocity (ms^-1^) | 0.70  (0.05) | 0.75  (0.05) | 0.87  (0.05) | 0.77  (0.05) | 0.165  [0.081, 0.249] | 0.036  [-0.044, 0.116] | **p = 0.048** | 0.817 |
| Cadence (steps/sec.) | 1.45  (0.05) | 1.54  (0.05) | 1.60  (0.05) | 1.56  (0.05) | 0.170  [0.085, 0.255] | 0.022  [-0.060, 0.104] | **p = 0.026** | 0.905 |
| Step length (metres) | 0.54  (0.02) | 0.51  (0.02) | 0.59  (0.02) | 0.53  (0.02) | 0.069  [0.036, 0.102] | 0.019  [-0.013,0.051] | **p = 0.051** | 0.793 |
| Stride length (metres) | 1.08  (0.05) | 1.02  (0.05) | 1.19  (0.05) | 1.06  (0.05) | 0.132  [0.069, 0.195] | 0.038  [-0.023, 0.099] | p = 0.057 | 2.28 |
| Stance phase (%) | 65.31  (1.00) | 64.52  (0.91) | 62.48  (1.00) | 64.20  (0.92) | -2.072  [-3.295, -0.848] | -0.925  [-2.128, 0.279] | p = 0.221 | 0.480 |
| Swing phase (%) | 34.69  (1.00) | 35.48  (0.91) | 37.52  (1.00) | 36.80  (0.92) | 2.072  [0.848, 3.295] | 0.925  [-0.279, 2.128] | p = 0.221 | 0.480 |
| Double support (%) | 30.62  (2.00) | 29.04  (1.83) | 24.96  (2.00) | 28.40  (1.85) | -4.143  [-6.590, -1.696] | -1.849  [-4.256, 0.558] | p = 0.221 | 0.537 |
| Single support (%)  Stride time variability | 66.38  (2.00)  138.33  (112.27) | 70.96  (1.83)  74.42  (40.71) | 72.04  (2.00)  75.14  (50.00) | 71.60  (1.85)  61.83  (36.40) | 4.413  [1.696, 6.590]  63.19  [-102.82, -23.56] | 1.849  [-0.558, 4.256]  -12.59  [-47.13, 21.94] | p = 0.221  p = 0.399 | 0.537  0.719 |

***Annex VIII:*** *Post-pre comparison: Spatiotemporal parameters of gait during dual tasking (performance of a serial subtraction task) on uneven surface*

SE = standard error, ^1^ Dance and control group were not significantly different for any measure at baseline, ^2^ Linear Mix Model applied for change scores. Covariates: gender and disease severity. Change score represents mean [95% CI], ^3^ Bold values are significant (*p*-value < 0.05), ^4^Effect sizes (d) calculated using Campbell Collaboration online calculator
